# Surface steric effect in heterogeneous catalysis as the origin of the high activity induced by strong metal-support interactions

**DOI:** 10.1016/j.isci.2025.112470

**Published:** 2025-04-17

**Authors:** Gerardo Valadez Huerta, Kaoru Hisama, Katsutoshi Sato, Katsutoshi Nagaoka, Michihisa Koyama

**Affiliations:** 1 Research Initiative for Supra Materials, Shinshu University, Nagano 380-8553, Japan; 2 Institute for Aqua Regeneration, Shinshu University, Nagano 380-8553, Japan; 3 Department of Chemical Systems Engineering, Nagoya University, Nagoya 464-8601, Japan; 4 Open Innovation Institute, Kyoto University, Kyoto 606-8501, Japan

**Keywords:** Chemistry, Catalysis

## Abstract

Supported nanoparticles offer unique opportunities for enhancing catalytic activity via strong metal-support interaction (SMSI). Even with state-of-the-art experimental techniques, the atomistic origin of this enhancement remains unclear, while current computational limitations make it difficult to provide a theoretical explanation. This study focused on clarifying the atomistic mechanism of SMSI by investigating N_2_ dissociation from Ru/La_0.5_Ce_0.5_O_1.75-x_ catalysts. Fast calculations using a neural network potential enabled the analysis of 328 complex nanoparticle models with varying degrees of site heterogeneity, encompassing over 25,768 adsorption sites. Our findings were validated against infrared spectra and helped identify catalyst configurations with enhanced catalytic activity, driven by SMSI. Specifically, the dissociation path of N_2_ molecules sandwiched between decoration cations on a nanoparticle near the support exhibited a low activation barrier. Our theoretical approach represents a major advancement in bridging the gap between simulation and empirical data and in our understanding of complex supported nanoparticle catalysts.

## Highlights

• 328 Ru/La_0_._5_Ce_0_._5_O_1_._75-x_ catalyst models generated with varying SMSI
• Over 25,000 N_2_-adsorption configurations calculated via neural network potential
• Reproduction of experimental IR spectra helped identify catalyst configurations
• Sandwiching by decoration cations due to SMSI enables fast N_2_ dissociation

## Graphical abstract

**Figure:**
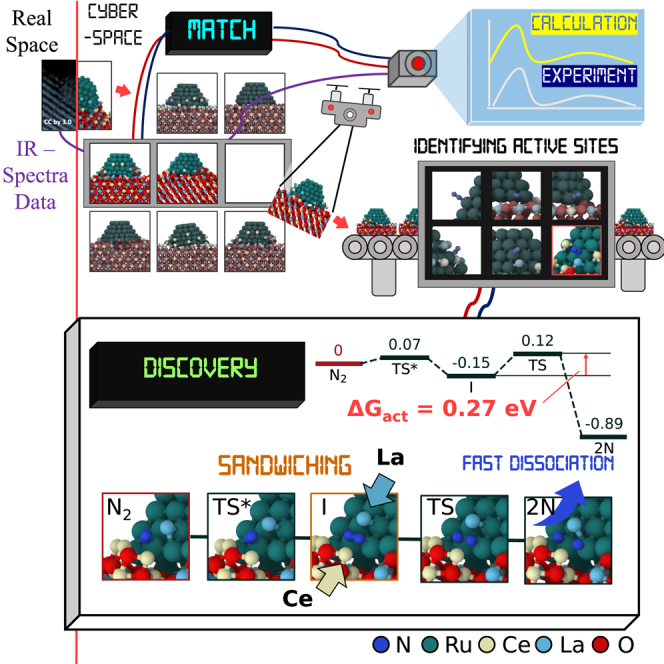

## Introduction

Heterogeneous catalysis plays a pivotal role in maintaining the current standards for human life, as it is used in approximately 80% of industrial chemical processes. This technology is indispensable for meeting basic needs and enhancing the quality of life. As we address concerns related to material circulation and environmental issues, the advancement of heterogeneous catalysis becomes even more imperative.^1^^,^^2^ However, conventional catalysts face inherent limitations determined by the main compositional element.^3^^,^^4^ The identification of new catalysts that can overcome these barriers is resulting in increasing catalyst complexity, ranging from single-atom catalysis and high-entropy materials to ligand-modified surfaces and catalysts assisted by external fields.^5^^,^^6^^,^^7^^,^^8^ Within this catalyst landscape, supported nanoparticles—metal nanoparticles dispersed upon a support—remain one of the most significant heterogeneous catalysis technologies.^9^^,^^10^ The impact of a support on nanoparticles was initially discussed in terms of stability and effective nanoparticle dispersion.^11^^,^^12^ However, since the discovery that a support can enhance the catalytic activity of nanoparticles,^11^^,^^13^ it has been assigned a higher value than being merely a carrier material.^12^

A strong support effect was first observed between titania and platinum-group metal nanoparticles that suppressed hydrogen adsorption, giving rise to the term “strong metal–support interaction” (SMSI).^11^^,^^14^^,^^15^ An attempt to explain SMSI was made by investigating the electronic effect of the support, which is characterized by charge transfer between the support and nanoparticles.^14^^,^^16^ However, the limitations of this approach became evident later as SMSI was observed to have a geometric nature.^16^^,^^17^ This steric effect is attributed to the decoration of metal sites by the support material. Such surface reconstructions not only inhibit adsorption sites,^11^^,^^14^ which have proven useful for selectivity tuning,^18^ but also create new catalytic sites with enhanced catalytic activity.^19^ Consequently, many catalysts that exploit SMSI have been developed in recent decades.^19^^,^^20^^,^^21^ Nevertheless, a complete theoretical explanation of the high activity of supported catalyst nanoparticles with SMSI is still lacking.^22^

Although the electronic and geometrical support effects have been measured^23^ and characterized experimentally,^24^ the atomistic mechanism underlying the high activity remains poorly understood, because the atomic arrangements contributing to this effect cannot be revealed. Theoretical studies are needed to address this problem. Existing computational approaches have examined electronic support effects without SMSI^24^^,^^25^ or simplified geometric effects computed on supported clusters decorated by a few atoms from the support.^26^^,^^27^ Nevertheless, computational studies cannot reproduce quantitative experimental data, because they are limited to a few sample systems^24^ due to the high computational cost of density functional theory (DFT) calculations, the workhorse for computational heterogeneous catalysis.^22^^,^^28^ This technique cannot be used to compute large models considering the various sites emerging due to the high heterogeneity of the catalyst surface,^29^ which is imperative for an accurate description of SMSI. As these issues remain unsolved, experimental and computational studies have failed to elucidate the mechanisms underlying SMSI, making it difficult to rationalize the sites contributing to the high activity.

This theoretical study elucidates the specific catalytic sites underlying the exceptional activity for N_2_ dissociation on Ru nanoparticles supported by La_0.5_Ce_0.5_O_1.75-x_. The catalytic activity for ammonia synthesis for catalysts reduced at 650°C (with a 43% reduction of Ce^4+^ to Ce^3+^) is comparable to that of Ru supported on Ca(NH_2_)_2_, which, to the best of our knowledge, is the catalyst with the highest reported activity.^23^^,^^30^ However, Ru/La_0.5_Ce_0.5_O_1.75-x_ demonstrates promising practicability compared to Ru/Ca(NH_2_)_2_, owing to its straightforward preparation process and superior chemical stability.^23^ The enhanced catalytic activity arises from the presence of an SMSI. The mechanism underlying this improvement has been attributed to the formation of an oxygen-deficient oxide layer on the surface of Ru nanoparticles, which exhibits strong electron-donating properties owing to its pronounced basicity.^31^ However, the precise atomic arrangement responsible for this phenomenon remains unknown. To elucidate the steric effect, we conducted an extensive computation involving over 25,768 adsorption sites using a universal neural network potential (UNNP).^32^ Our method considered 328 complex models of supported nanoparticles, showing wide degrees of site heterogeneity arising from SMSI. The robustness of our findings is evidenced by the statistical recreation of infrared (IR) spectra of N_2_ adsorption, which demonstrate exceptional precision. This bridging of simulation results and empirical data enabled us to identify configurations of catalysts demonstrating elevated activity enhanced by SMSI. Our computational findings revealed the presence of N_2_ molecules undergoing dissociation, with the molecules sandwiched between Ce and La cations on a nanoparticle near the support. Notably, adsorbed molecules showing this geometrical feature with corresponding low-wavenumber features in their IR spectra can result in a remarkably low activation barrier of 0.27 eV, offering a promising possibility for catalytic enhancement within a rational catalyst design strategy.

## Results

### Strategy and catalyst model preparation

**Figure 1:**
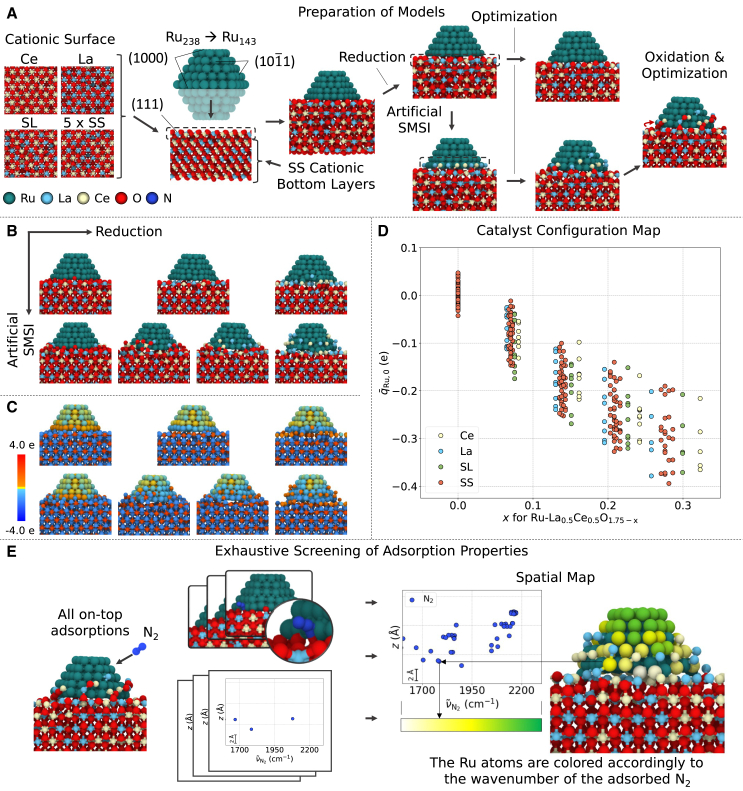
Strategy for modeling and simulating N_2_ dissociation on Ru/La_0.5_Ce_0.5_O_1.75-x_ catalysts (A) Automated preparation of the models. (B) Optimized catalyst configurations for three reduction degrees of the cationic surface and two artificial SMSIs. (C) Charge distribution for the configurations in (B). (D) Catalyst configuration map spread by the average charge *q*_Ru,0_ of Ru atoms on the nanoparticle surface layer and reduction degree *x* for all 328 catalyst configurations analyzed in this study (*x* corresponds to the composition of the 4 × 4 × 3 La_0.5_Ce_0.5_O_1.75-x_ slab). (E) Screening method used to calculate the N_2_ properties and create the spatial maps. The N_2_ wavenumber ${\tilde{\nu }}_{N_{2}}$ for each N_2_ adsorption state was plotted over the position of the Ru site for each configuration. The Ru sites were then colored according to the value of the wavenumber. The coordinate z is given arbitrarily for the Ru sites.

Our approach for modeling and simulating N_2_ dissociation on supported Ru nanoparticles is depicted in Figures 1 and S1. First, we modeled the La_0.5_Ce_0.5_O_1.75-x_ oxide support. La_0.5_Ce_0.5_O_1.75_ stabilizes by forming a solid-solution (SS) from the composite oxides CeO_2_ and La_2_O_3_ in the cubic fluorite structure (space group, Fd $\bar{3}$ m) with Ce^4+^ and La^3+^ cations, oxygen anions and, oxygen vacancies (Data S1).^23^ Several 4 × 4 × 3 La_0.5_Ce_0.5_O_1.75_(111) slab models were developed. We refer to the upper layer of the cationic slab as the cationic surface hereafter. We prepared Ce, La, superlattice (SL), and five SS cationic surfaces (Figure 1A). The SL surface was prepared by intercalating the Ce and La cations and SS surfaces via random distribution. The remaining cationic bottom layers were modeled as an SS phase. Ru/La_0.5_Ce_0.5_O_1.75_ models were prepared by placing a Ru_143_ half-nanoparticle on the La_0.5_Ce_0.5_O_1.75_ slab (Figure 1A). After catalyst reduction, the oxide support’s composition becomes thermostabilized to La_0.5_Ce_0.5_O_1.75-x_.^33^ Accordingly, we reduced the slab by removing oxygen atoms from the cationic surface after assembling the half-nanoparticle and support models, referring to the *x* in the final La_0.5_Ce_0.5_O_1.75-x_ slab composition as the reduction degree (Data S1). The onset of an SMSI was observed experimentally for this particular catalyst,^23^ indicating an increase in the nanoparticle-metal oxide interfacial area.^34^^,^^35^ To model the catalyst structure with an artificial SMSI, we randomly selected cations from the cationic surface and placed them on the lowest nanoparticle layer. Catalyst configurations were obtained by optimizing the models with different degrees of cationic surface reduction and artificial SMSI (Figure S2). Additional configurations were generated by relocating oxygen atoms from the cationic surface to the cations decorating the nanoparticle after relaxation. During this process, the anion-to-cation ratio at the encapsulation and the cationic surface was kept equal. Thus, the final catalyst configurations included nanoparticle encapsulation models both with and without oxygen relocation.

We observed different degrees of SMSI onset, depending on the extent of cationic surface reduction and artificial SMSI (Figure 1B and Data S1). Without reduction, no nanoparticle encapsulation could be observed. Only for surfaces with full reduction, where all oxygen atoms in the surface layer were removed, the surface reconstruction after optimization resulted in only one or two cations moving toward the upper layers of the Ru nanoparticle without the artificial SMSI. The observed surface reconstructions did not stabilize oxygen atoms on the upper layers of the Ru nanoparticle. When more cations are initially placed on the nanoparticle at a high reduction degree, they remain at this position or move toward the upper layers. In contrast, rather than having oxygen atoms move up to encapsulate the nanoparticle, all cations return to the support if no reduction is applied. Between these two limiting cases, some of the initial cations contributed to the encapsulation of the nanoparticle’s upper layers as single atoms or by forming local arrangements with oxygen anions, while the rest returned to the cationic surface. Interestingly, if oxygen anions were relocated to the encapsulation, the oxidized cations remained in their positions rather than returning to the support, forming an anion-cation association. We observed the nanoparticle beginning to convulse in configurations with high SMSI and high reduction degree *x* (Figure S3). This type of system deterioration resulting from a high SMSI is an experimentally observed process.^35^

Next, we mapped the optimized catalyst configurations to obtain a complete picture of the configurational space. For this purpose, we needed suitable descriptors. As widely discussed,^36^^,^^37^ the electronic metal-support interaction (EMSI) is suitable for describing the catalyst support effect. The EMSI describes the charge transfer between a nanoparticle and its support. Therefore, it can be represented by the average charge of Ru. The charge distribution of Ru atoms and, thus, their average charge shift toward more negative values, depending on the degree of cationic surface reduction and SMSI (see blue colored atoms in Figure 1C). Thus, we used the average charge ${\bar{q}}_{\text{Ru},0}$ of the Ru atoms at the surface nanoparticle layer and the value of *x* in La_0.5_Ce_0.5_O_1.75-x_ to distribute the Ru/La_0.5_Ce_0.5_O_1.75-x_(111) optimized configurations. The configuration map in Figure 1D was constructed according to these descriptors. Using different upper cationic slab layer compositions and reduction degrees, as well as artificial modeling of the SMSI, we obtained 328 catalyst configurations. The maximum experimentally observed reduction degree is 0.25. In Data S1, we thoroughly discuss that the defined x does not represent the actual reduction degree, as it tends to be overestimated due to the finite slab size. While we consider all configurations to discuss the resulting configurational maps of catalyst and adsorption states, configurations with *x* > 0.25 were excluded when addressing real systems to be certain of modeling realistic compositions.

Finally, we accounted for N_2_ activation. For each of the 328 catalyst configurations, we placed a N_2_ molecule with an end-on orientation on each available Ru on-top site by referring to previous experimental and theoretical observations of the adsorption structure.^23^^,^^38^ This step resulted in 25,768 optimizations. To work with this massive amount of data, we scanned, evaluated, and plotted the properties for each adsorption state using an automated screening approach (Figure 1E). This step enabled us to automatically create spatial maps for each catalyst configuration by assigning, for example, specific N_2_ wavenumber values to the Ru site position (compare Figures 1E and S4).

### Exhaustive calculation results of N_2_ adsorption

**Figure 2:**
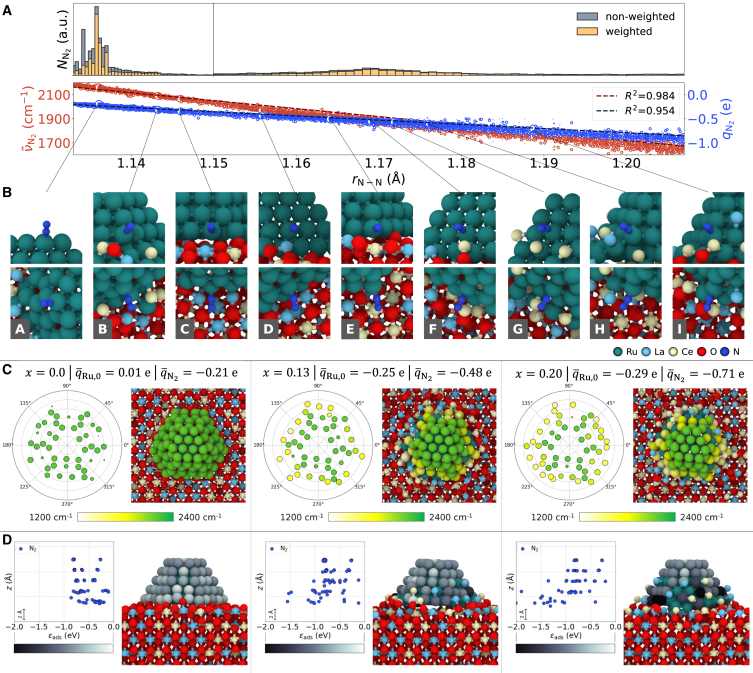
Simulated N_2_ adsorption on different Ru/La_0.5_Ce_0.5_O_1.75-x_ catalyst configurations (A) Top: weighted and non-weighted histograms of the N–N bond length *r*_N-N_ distribution. A total of 50 bins for 1.13 Å ≤ *r*_N-N_ < 1.15 Å and 150 bins for 1.15 Å ≤ *r*_N-N_ < 1.30 Å were arbitrarily chosen. The N_2_ coverage *θ* at 25°C and 6 kPa were used as the weighting factors. Bottom: simulated wavenumbers ${\tilde{\nu }}_{N_{2}}$ and charges *q*_N2_ of single adsorbed N_2_ molecules for all adsorption sites within the depicted range. (B) Representative adsorption sites. (C) Calculated spatial maps of the N_2_ wavenumber, which were built as described in Figure 1E. Here, the top view and polar coordinates of the catalyst were used. The diameter of the markers is given according to the N_2_ coverage *θ* at 25°C and 6 kPa. (D) Spatial maps of the N_2_ adsorption energy *ε*_ads_, which were built as described in Figure 1E.

Histograms showing the distribution of all calculated N_2_ adsorption states over the N–N bond length are depicted in Figure 2A. The coverage at 25°C and 6 kPa was used as the weighting factor for the weighted histogram. Two dense regions with bond length peaks of approximately 1.14 and 1.17 Å were identified. Two adsorption peaks at approximately 2,150 and 1,850 cm^−1^, corresponding to two N–N stretching vibrational modes with a high and a low wavenumber, respectively, are also observed (see bottom diagram in Figure 2A). The high-frequency peak arises from similar adsorption configurations at the uppermost layer of the nanoparticle across all models, while the low-frequency peak suggests frequently occurring adsorption states due to SMSI. Nevertheless, the distribution shape and features reflect the range of possible configurations and may vary with the modeling approach.

Spatial maps of the N_2_ wavenumber are illustrated in Figure 2C. Here, we used the top view of the nanoparticles and polar coordinate systems and kept the sizes of the markers proportional to the simulated N_2_ coverage *θ*(*T* = 25°C, *p* = 6 kPa) for better comparison. The adsorbed N_2_ molecules had a high wavenumber (> 2,000 cm^−1^) when *x* ≈ ${\bar{q}}_{Ru,0}$ ≈ 0. As *x* increased and the EMSI became stronger, a lower wavenumber (< 2,000 cm^−1^) was observed. Sites with a low wavenumber were located near Ce and La cations wrapped around the nanoparticle. Furthermore, adsorption sites became more active at higher *x* and EMSI, as indicated by the size of the markers. To explain this effect, we constructed spatial maps of the N_2_ adsorption energy, as shown in Figure 2D. For configurations with *x* ≈ ${\bar{q}}_{\text{Ru},0}$ ≈ 0, the adsorption on the edge and vertex sites is stronger than that on the ($10\bar{1}1$) and (0001) facets of the nanoparticle (see Figure 1A for facet orientations). As *x* and EMSI increased, the adsorption energy decreased and, thus, *θ* increased at sites near Ce and La cations. When the non-weighted and weighted distributions of the histograms in Figure 2A are compared, the activation of sites with a lower wavenumber become more evident. Moreover, as the number of cations wrapping the nanoparticle increased, the Ru sites became increasingly occupied, thereby preventing the adsorption of N_2_ molecules (Figure 2C, ($10\bar{1}1$) surfaces for the catalyst configuration with *x* = 0.20 and ${\bar{q}}_{Ru,0}$ = −0.29 e). Finally, the wavenumber and N_2_ molecular charge showed a linear relationship with the N_2_ bond length (bottom diagram in Figure 2A). The N–N bond became weaker owing to electron transfer to the N_2_ antibonding *π*-orbital.^39^ While our calculations effectively reproduced this trend, we stress that this statement is simply an interpretation of the results because the UNNP model does not consider electron transfer explicitly.

After performing the exhaustive scan described previously, we searched for representative adsorption states. A representative adsorption state should have high coverage and have a statistical bulk density within two defined regions. Figure 2B shows examples of adsorption states following these criteria (A–I). N_2_ molecules adsorbed on the upper layers of the nanoparticle showed a high wavenumber. For example, the peak for adsorption state A exhibits a wavenumber of 2,161 cm^−1^. Two main mechanisms that determine the extent of the decrease in the wavenumber were identified. The first effect is applicable to molecules adsorbed near decoration cations on the nanoparticle (e.g., states B, D, F, G, and H). When many cations are present near the adsorption site, this effect is enhanced. However, when the cations are (partially) oxidized, the effect is dampened (compare F and G). The second mechanism is applicable to molecules adsorbed at the interface between the nanoparticle and support, specifically at the lowest nanoparticle layer (adsorption states C and E). In this case, a lower wavenumber is observed even without cations near the adsorption site. However, this finding applies only to supports with a non-zero *x* or interfacial regions with oxygen vacancies when *x* is zero. When both mechanisms are combined, wavenumbers below 1,800 cm^−1^ are observed, even when the cations attached to the nanoparticle are partially oxidized, as observed for adsorption state I.

### Catalyst configuration of a real system

**Figure 3:**
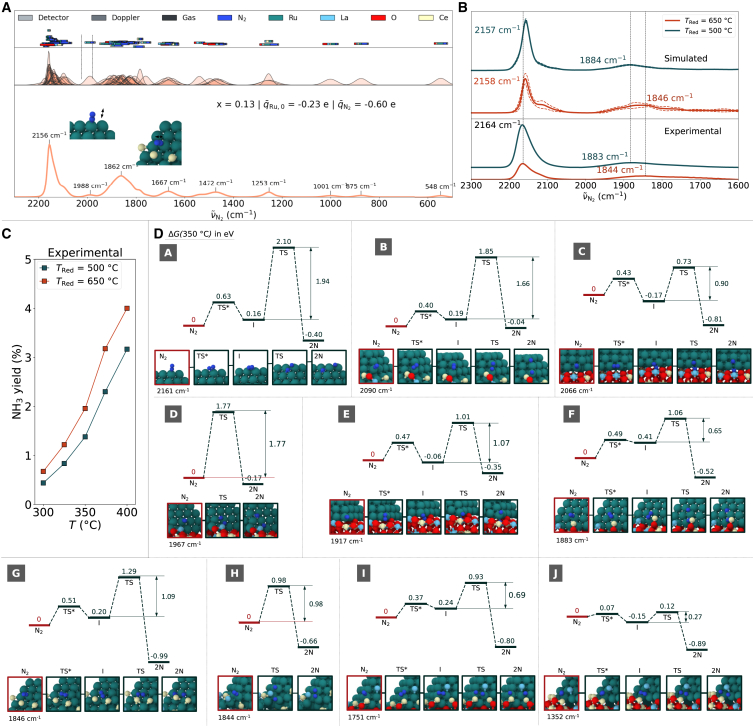
Simulated catalytic properties for N_2_ dissociation on different Ru/La_0.5_Ce_0.5_O_1.75-x_ catalyst configurations (A) Example of a simulated N_2_ wavenumber distribution. Top: Calculated spectral line widths colored according to the calculated contributions: broadening due to the detector resolution, Doppler effect, and collisions with the N_2_ gas atmosphere, neighboring Ru,Ce,La and O atoms, and adsorbed N_2_. Middle: Weighted Gaussians (weighting factor: N_2_ coverage *θ*). Bottom: Wavenumber distribution obtained from the sum of the Gaussians. The values were obtained at 25°C and 6 kPa. (B) Calculated average N_2_ wavenumber distributions and measured IR spectra.^33^ Averaging was performed over the distributions that are statistically similar to the experimental IR spectra.^33^ Data are represented as mean ± SEM. (C) Measured NH_3_ yield (%) as a function of the reduction temperature of the catalysts.^23^ (D) Calculated N_2_ dissociation paths for the adsorption states A to I shown in Figure 2B. An extra dissociation path J with a low dissociation barrier of 0.27 eV is also depicted. For each path, the label N_2_ describes the initial state, TS∗ and TS indicate the transition states, I indicates the intermediate state, and 2N indicates the final dissociation state. The energy values indicated correspond to the Gibbs energy difference Δ*G*(350°C) relative to the initial state. The energies for the rate-determining step are also provided. All experimental data were taken from the literature.^23^

Further validation is required to identify a catalyst configuration representing a real system. Thus, we calculated the wavenumber spectrum for each catalyst configuration by estimating weighted Gaussians for each adsorption state (middle diagram in Figure 3A) and compared the results with experimental IR spectra. Only configurations with x ≤ 0.25 were considered. The peak broadening of each vibrational state was accounted using the actual detector resolution, Doppler effect, and collisional linewidth. The collision contributions consider the gas atmosphere under experimental conditions, surrounding catalyst atoms, and neighboring adsorbed N_2_ molecules. The coverage at 25°C and 6 kPa was used as a weighting factor. The contribution of each effect is shown in the top diagram of Figure 3A. The spectrum of the wavenumber of the catalyst configuration was obtained by summing the Gaussians of each adsorption configuration (Data S2).

We identified regions with higher and lower wavenumbers using different distributions. In general, the shape of a distribution follows a single Gaussian. Lower coverage or higher broadening results in lower peaks; higher broadening also leads to a wider distribution. Because the simulated spectrum represents the sum of the Gaussians, the height of the spectrum increases with the number of N_2_ molecules with wavenumbers close to a specific value. In the simulated spectra (e.g., bottom diagram in Figure 3A), the broadening of the high wavenumber region can simply be represented by the collisions with the Ru site and adjacent N_2_ molecules, as the Doppler and gas atmosphere effects are negligible. The asymmetry observed in this case is due to the wide distribution of adsorption states with wavenumbers of 2,000–2,100 cm^−1^. The relatively low coverage within this wavenumber interval (see histograms in Figure 2A) results in Gaussians with low peaks. The high number of neighboring collisions in the low-frequency region causes a larger broadening of the calculated distribution compared with that observed in the high-frequency region. Moreover, the distribution curve around the high-frequency peak flattens as the distribution in the low-wavenumber region rises. This effect is consistent with the activation of adsorption sites within the low-frequency region discussed in the previous section.

The simulated spectra were compared with the baseline corrected IR spectra of catalysts reduced at a reduction temperature *T*_Red_ of 500°C and 650°C.^23^ Accordingly, 40 and 7 of the 328 simulated spectra were statistically similar to the experimental spectra for *T*_Red_ = 500°C and 650°C, respectively (Data S3). The properties of the catalyst configuration sets are discussed in Data S4. The average of these spectra, together with the experimental spectra, is provided in Figure 3B. The average spectrum corresponding to the catalyst reduced at 500°C shows a peak at a low wavenumber of 1,884 cm^−1^. The average spectrum corresponding to the catalyst reduced at 650°C shows a peak at a low wavenumber of 1,846 cm^−1^. Thus, the measured values of 1,883 and 1,844 cm^−1^ were reproduced by the model, where all identified peaks correspond to frequencies with stretching vibrational modes. We further calculated the relative peak intensity by dividing the peak intensities at high wavenumbers by those at low wavenumbers. The simulated and experimental relative intensities were 8.83 and 9.06, respectively, for *T*_red_ = 500°C, and 4.47 and 4.43, respectively, for *T*_Red_ = 650°C, showing very good agreement. Because the shape of the calculated wavenumber distribution effectively reproduces the experimental spectra, the experimental observations can be analyzed based on a physical background.

### Analysis of catalytic activity

In experiments, Ru/La_0.5_Ce_0.5_O_1.75-x_ catalysts show maximum activity when reduced at 650°C (Figure 3C).^23^ To elucidate the physical origin of this phenomenon, we calculated the dissociation paths (Figure 3D) for the N_2_ adsorption states shown in Figure 2B at 350°C. Given that the activation barrier for N_2_ dissociation is by far higher than that of other steps in ammonia synthesis, we can simplify the kinetics by assuming a single rate-determining step.^40^ The activation Gibbs energy for the rate-determining step of dissociation on top of the nanoparticle (state A) is 1.94 eV. This value is comparable with the reported energy barrier for N_2_ dissociation on a Ru slab (Table S1). The activation Gibbs energies for adsorption states B to E, and G and H, range from 0.90 eV to 1.85 eV. Lower values of 0.65 and 0.69 eV are observed for states F and I, respectively. These active sites F and I are located close to the interface, where the reduction affects the structure and adsorption state of N_2_ (Figure 2C) and stabilize the onset of an SMSI. A correlation between the activation Gibbs energies and the wavenumbers could not be observed.

**Figure 4:**
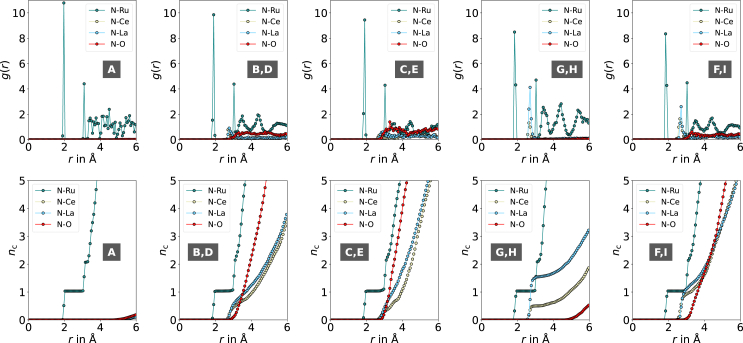
Simulated RDFs partial RDFs *g(r)* and average coordination numbers *n*_*c*_ calculated from averaging sites with similar geometrical catalytic activity as categorized in Table 1

We further performed structural analysis to clearly differentiate these adsorption states (see Data S5). The number of surrounding Ce, La, and O (*N*_Ce+La_ and *N*_O_) atoms near the N_2_ molecule, the distance between the N_2_ molecule and the support, and the wavenumber range were sufficient to be used as descriptors. To demonstrate the effectiveness of our categorization, we computed the partial radial distribution function *g(r)* for all adsorption states within each category, along with the average nearest neighbor count *n*_*c*_ (see Figure 4). Adsorption states with a high catalytic activity, akin to F and I, typically exhibit coordination with a Ru, Ce, and La atom (see Figure S5 for additional examples). This unique geometric arrangement sets them apart from other adsorption states (Figure S6). What these adsorption states have in common is that the N_2_ molecule is adsorbed end-on at a site on the nanoparticle, enabling access to a position where it is sandwiched between a La and Ce cation during dissociation. To further illustrate the effect of this specific geometry on the catalytic activity, we selected one of the adsorption states with a lower wavenumber of 1352 cm^−1^ that displayed this geometrical feature and calculated the dissociation barrier (see Figure 3D, dissociation path J). As observed, the end-on site initially stabilizes into a side-on site and then proceeds to dissociate, overcoming an exceptionally low barrier of 0.27 eV, supporting our claims. These types of adsorption states present a significant opportunity for further catalytic activity enhancement through the adjustment of SMSI within a rational catalyst design strategy targeting this specific configuration. Finally, we clarified whether our hypothesis can explain the measured catalytic activity of the catalysts (Figure 3C). We counted the average number of states F and I for each configuration corresponding to T_Red_ = 500°C and T_Red_ = 650°C considering the N_2_ coverage at 350°C and 1 MPa (Table 1). On average, we observed 2.36 times more of these states in catalyst configurations corresponding *T*_Red_ = 650°C compared with those corresponding *T*_Red_ = 500°C, thus supporting the higher activity of the catalyst system reduced at 650°C (see Data S6 for further discussion).

**Table 1:** Average number of adsorption states for the simulated configurations assigned to real catalysts

States	A	B	D	C	E	G	H	F	I
ΔG(350°C) (eV)	1.94	1.85	1.77	0.90	1.01	1.09	0.98	0.65	0.69
${\bar{N}}_{N_{2}}\left(T_{\text{red}}=500\;{}^{\circ}C\right)$	17.28	2.36		0.36		0.83		1.63	
${\bar{N}}_{N_{2}}\left(T_{\text{red}}=650\;{}^{\circ}C\right)$	12.54	1.42		0.0		2.35		3.84	

ΔG(350°C) is the activation barrier at 350 °C. ${\bar{N}}_{N_{2}}\left(T_{\text{red}}\right)$ is the average weighted number of adsorption states corresponding to the states A, C, G, H, F, and I (Figure 1D) for the configurations assigned to the catalyst at a reduction temperature of *T*_red_. The N_2_ coverage *θ*(*T* = 350 °C and *p* = 1 MPa) was used as the weighting factor.

## Discussion

The findings of this theoretical investigation effectively revealed the key geometric features that define the catalytic sites primarily responsible for the elevated activity driven by SMSI in the context of N_2_ dissociation on Ru nanoparticles supported by La_0.5_Ce_0.5_O_1.75-x_. In the proposed framework, the modeling approach for the catalyst is more compliant with an artificial mapping of configurations than a completely physics-based approach, i.e., guided by the chemical potentials of the components and reducing atmosphere. This mapping was necessary to achieve the adequate and systematic preparation of catalyst models used to explore the adsorption and catalytic properties of molecules with which they interact. The exhaustive calculation of adsorbed molecules on the catalyst was unavoidable. The massive size of our models and extensive surface reconstruction necessary during the long optimization of the supported nanoparticle systems prohibit the use of first-principles calculations. Even if optimizing some adsorption sites via this technique is possible, the results obtained cannot be assumed to be meaningful for such a complex heterogeneous system, as discussed throughout the article. Moreover, we successfully validated the numerical results with experimental data and clarified specific experimental observations for these types of catalysts. Notably, we provided experimental observations for the IR spectra of N_2_ molecules adsorbed on the studied catalyst with a robust physical background, which may be applicable to other similar catalysis systems.

Achieving the number of calculations required to obtain the dissociation reaction paths similar to that employed for the 25,768 calculated adsorption sites in our work will be challenging. However, we can produce an accurate state-of-the-art approximation of such properties for the heterogeneous catalyst system studied here. In particular, we elucidated the geometric features of the catalytic sites responsible for the heightened catalytic activity, i.e., the unique geometry that enables the sandwiching of N_2_ molecules between La and Ce cations during dissociation. If the distribution of cations around a nanoparticle can be skillfully controlled by modifying the nanoparticle composition to avoid the deactivation of strategic adsorption sites and targeting the optimal geometric configurations, new catalysts with higher catalytic activity could be designed, synthesized, and characterized experimentally for use in practical systems.

The mechanisms that contribute to a high activity may vary depending on the catalyst system, owing to the inherent complexity of SMSI. However, our approach can be directly used to investigate other material combinations, including other supports and nanoparticles based on binary, ternary, and multinary alloys. The many unresolved questions arising from the unique properties and behaviors of the innumerable complex supported nanoparticle catalysts synthesized to date could be elucidated using the proposed framework. Accordingly, we view this study as an essential initial step toward the systematic design and rationalization of supported nanoparticles showing SMSI, offering a promising avenue for further advancements in the field.

### Limitations of the study

While certain limitations of the study have been discussed throughout the main text and the supplemental information, additional important limitations are summarized here. The analysis was limited to single-adsorption configurations, and, thus, co-adsorption of multiple N_2_ molecules or other reaction intermediates was not considered. Furthermore, the study focuses on N_2_ dissociation as the rate-determining step in ammonia synthesis, while the steric effect on other elementary reaction steps was not explicitly addressed. Although agreement with IR spectra supports the identified catalyst configurations, direct validation using atomic-resolution microscopy is still lacking. Finally, as the electronic structure was not calculated, the electronic effect induced by SMSI on the catalytic activity remains an open question for future investigation.

## Resource availability

### Lead contact

Further information and resource requests should be directed to the lead contact, Gerardo Valadez Huerta (valadez@shinshu-u.ac.jp).

### Materials availability

No physical materials, chemicals, or biological reagents were used or generated in this study.

### Data and code availability

• Data: Additional data supporting the findings and statements presented in this study can be found in the supplemental information and Supplemental Data Files A and B (see Data S7). The Data were deposited on Mendeley Data at https://doi.org/10.17632/w7xjrr4fc3.1.
• Code: The code used in this study is available in the “Code” directory within the Mendeley Data repository at https://doi.org/10.17632/w7xjrr4fc3.1.
• Other: Additional data or materials can be obtained from the lead contact upon reasonable request.

## Acknowledgments

This work was supported by the Alexander von Humboldt Stiftung (P20701) and 10.13039/501100001691Japan Society for the Promotion of Science (10.13039/501100001691KAKENHI grant Nos. JP21F30701 and JP20H05623). The authors are especially grateful to the participants of the Data-Driven AI Laboratory of the Research Initiative for Supra Materials at Shinshu University for their comments and suggestions during the whole project. All images of the atomic models within the article were rendered using OVITO.^41^ The HAADF-STEM image included in the Graphical Abstract is reproduced from Y. Ogura, K. Sato, S. Miyahara, Y. Kawano, T. Toriyama, T. Yamamoto, S. Matsumura, S. Hosokawa, and K. Nagaoka, Chem. Sci., 2018, 9, 2230 https://doi.org/10.1039/C7SC05343F with permission from the Royal Society of Chemistry.

## Author contributions

Conceptualization, G.V.H., M.K., K.N., and K.S.; methodology, G.V.H.; software, G.V.H.; validation, G.V.H.; formal analysis, G.V.H. and K.H.; investigation, G.V.H.; resources, M.K.; data curation, G.V.H.; writing – original draft, G.V.H.; writing – review and editing, G.V.H, M.K., K.H., K.N., and K.S.; visualization, G.V.H.; supervision, M.K. and K.N.; project administration, M.K.; funding acquisition, G.V.H. and M.K.

## Declaration of interests

The authors declare no competing interests.

## STAR★Methods

### Key resources table

**Table:** 

REAGENT or RESOURCE	SOURCE	IDENTIFIER
**Deposited data**		

Data SA	This Study	https://doi.org/10.17632/w7xjrr4fc3.1
Data SB	This Study	https://doi.org/10.17632/w7xjrr4fc3.1
Code	This Study	https://doi.org/10.17632/w7xjrr4fc3.1
Materials Project Database	Jain et al.^42^	https://next-gen.materialsproject.org/

**Software and algorithms**		

PreFerred Potential version 1.0.0	Matlantis^32^	https://matlantis.com/
Atomic Simulation Environment	Larsen et al.^43^	https://wiki.fysik.dtu.dk/ase/
FLARE	Vandermause et al.^44^	https://flare.readthedocs.io/
OVITO	OVITO Gmbh^41^	https://www.ovito.org/
Python version 3.9	Python^TM^	https://www.python.org/
MS Word	Microsoft Corporation	https://www.microsoft.com/en-us/microsoft-365/word
MS PowerPoint	Microsoft Corporation	https://www.microsoft.com/en-us/microsoft-365/powerpoint
MS Excel	Microsoft Corporation	https://www.microsoft.com/en-us/microsoft-365/excel

### Method details

#### Model preparation and calculation

We configured the 4 × 4 × 3 La_0.5_Ce_0.5_O_1.75_ bulk models prior to optimization using a well-known method for binary metal-oxides with vacancies^45^ by accounting for a Warren–Cowley^46^ parameter close to zero for the SS cationic phase and a lattice parameter of 7.93 Å corresponding to the DFT values presented in the Materials Project Database.^42^ Energy minimization was applied to the cell size and atom positions. After optimization, we added a 30 Å vacuum layer perpendicular to the (111) surface to create a slab and then optimized the atomic positions. We conducted a molecular dynamics simulation on the canonical ensemble to equilibrate the anions of the slab at 650°C, corresponding to the reduction temperature reported in the literature^23^ (Figure S29). The configuration of the slab trajectory obtained after equilibration was used for further calculations, and the upper cationic layer was reduced by different degrees by randomly removing oxygen anions.

We optimized a Ru_238_ nanoparticle with a hexagonal close-packed structure and extracted a Ru_143_ half-nanoparticle with a large (0001) facet downside. We tested different orientations of the nanoparticle on the slab and chose the most energetically favorable one (Methods S1). Different supported nanoparticle configurations were created using this nanoparticle orientation. We then placed random cations from the cationic surface at the lower nanoparticle layer at various degrees and optimized the structure. Individual N_2_ molecules were optimized and placed at all Ru on-top sites at a distance of 1.6 Å. The N atoms were oriented so that they were aligned parallel along the direction from the Ru site to the middle of the nanoparticle (Figure S1). After optimization, further evaluation was performed using only molecules that did not change the Ru site and with a distance to this site of less than 2.3 Å, bond length less than 2.5 Å, and wavenumber higher than 1,200 cm^−1^. These criteria ensured that the molecules were end-on adsorbed on Ru on-top sites without repetition.

To approximate the configurations for two different dissociated states per adsorption site, we placed N atoms at the neighboring site of Ru in the $R^{2}$ projection of the nanoparticle surface layer and optimized them. Saddle-point configurations were calculated using two configurations estimated as the linear interpolation with a factor of 0.5 between the initial state and two approximated final states. Ten saddle points were calculated for each configuration. A second configuration was minimized downhill by extrapolating it 105% away from the initial state toward the saddle point and conducting a final optimization to find the nearest local minima. Then, the minimum energy path was calculated, disregarding the reaction paths that were repeated or inconsistent. Non-consistent paths were defined as those with energies higher than the calculated transition state, energy barriers higher than 4 eV, a non-dissociated final state, or a poorly converged transition state showing more than one imaginary frequency.

#### Simulation methods

All simulations were conducted using the commercial UNNP Preferred Potential within the Matlantis distribution.^32^ The UNNP corresponds to the pre-trained version 1.0.0. The UNNP, trained on DFT data with high accuracy for forces and energies, correctly accounts for local oxidation states, meaning ionization is inherently considered during calculations. Partial charges from Bader analysis are obtained via a separate neural network and, by predicting their DFT counterparts, generally follow the trend but do not necessarily correspond to expected valences, nor do they influence energy or forces. Although Matlantis includes numerous features, we used the freely available Atomic Simulation Environment (ASE)^43^ tools for all calculations. All-atom energy optimizations were performed using the Fast Inertial Relaxation Engine (FIRE) algorithm^47^ with a force threshold of 0.001 eV/Å unless otherwise stated. The molecular dynamics simulation was performed with a timestep of 0.5 fs. The Nosé–Hoover^48^ thermostat, as implemented in the Fast Learning of Atomistic Rare Events (FLARE) library,^44^ was used with a damping factor *Q* corresponding to 100 timesteps.^49^ A simulation time of 100 ps was sufficient to achieve equilibration. The Dimer method^50^ was used to search for saddle points. We performed downhill optimization using the Broyden–Fletcher–Goldfarb–Shanno line search method.^43^ Only atoms within a cut-off radius of 3.0 Å relative to the N atoms were allowed to move. The same was true for the Nudged Elastic Band (NEB) routine,^51^ that was used to search for the minimum energy path with a force threshold of 0.05 eV/Å. The NEB routine was performed by separately calculating segments of three images and adding in-between configurations along the path, resulting in nine images after two iterations, including the initial, transition, and final states. The NEB calculation was repeated for segments with a local maximum by climbing the images^52^ with a force threshold of 0.005 eV/Å. Configurations corresponding to the local minimum within the path were further all-atom optimized. A spring constant of 0.1 eV/Å was used for all NEB calculations. UNNP validation is described in Methods S2.

#### Calculation of thermophysical properties

We calculated the coverage *θ* assuming Langmuir adsorption as *θ = K*_ads_⋅*p*/(1 + *K*_ads_⋅*p*) with the equilibrium constant *K*_ads_ calculated from ln(p^o^⋅*K*_ads_) = -Δ*G*(*T*,*p*)/(k_B_*T*). Here, *p* is pressure, *p*^o^ is standard pressure, *T* is temperature, and *k*_B_ is Boltzmann’s constant. The adsorption Gibbs energy Δ*G*(*T*,*p*) for a single N_2_ molecule was calculated by assuming the ideal gas limit, and the Gibbs energy for the adsorbed molecule was approximated using the Helmholtz energy from the harmonic limit; here, both models were used as implemented in ASE.^43^

To calculate the wavenumber spectrum (Figure S30), we determined the Doppler broadening $\delta {\tilde{\nu }}_{D}$ as follows^53^:

$$\delta {\tilde{\nu }}_{D}=\frac{2\nu }{c}{\left(\frac{2\cdot k_{B}T\;\ln\left(2\right)}{m_{N_{2}}}\right)}^{\frac{1}{2}}$$

where *ν* denotes the wavenumber, *c* is the speed of light in vacuum, and $m_{N_{2}}$ is the mass of the N_2_ molecule. The broadening due to collisions was given by^53^:

$$\delta {\tilde{\nu }}_{C,i}=\frac{\pi r^{2}}{2c}{\left(\frac{8k_{B}T}{\pi \mu_{N_{2}-i}}\right)}^{\frac{1}{2}}\cdot \rho_{i}$$

where the reduced mass *μ*_(A-B)_ = *m*_A_ *m*_B_/(m_A_ + *m*_B_) and *ρ*_i_ is the number density of component *i* corresponding to the ideal gas density for collisions with the N_2_ atmosphere or the number of atoms/molecules within the volume of the sphere with a radius equal to the collision cross-section *πr*^2^ = 3.4 nm^2^ for N_2_ molecules.^53^ The density was weighted using *θ* by accounting for possible adjacent adsorbed N_2_ molecules. Finally, we considered the detector resolution by applying the Sparrow rule^54^ as follows:

$${\delta \tilde{\nu }}_{\text{Res}}=\sqrt{2\;\ln\left(2\right)\;}\cdot \text{FWHM,}$$

where the full width at half maximum (FWHM) for each Gaussian is given by the experimental resolution^23^ of 4 cm^−1^.

### Quantification and statistical analysis

The uncertainty of average values is estimated using the standard error of the mean (SEM) at a 95% confidence interval and is provided in parenthetic notation^55^ or depicted as error bars in diagrams. Equilibration of molecular dynamics simulations was evaluated using the method of Chodera.^56^
