# Supplementary material for: Surface steric effect in heterogeneous catalysis as the origin of the high activity induced by strong metal-support interactions
Source: iScience. 2025 Apr 17;28(5):112470. doi: 10.1016/j.isci.2025.112470 (PMC12124641; doi:10.1016/j.isci.2025.112470)
Supplement: Document S1. Data S1–S7, Methods S1–S5 Figures S1–S32 and Table S1–S14 [file mmc1.pdf]

iScience, Volume *28*

Supplemental information

Surface steric effect in heterogeneous catalysis as the origin of the high activity induced by strong metal-support interactions

Gerardo Valadez Huerta, Kaoru Hisama, Katsutoshi Sato, Katsutoshi Nagaoka, and Michihisa Koyama

Data S1: **Further Discussion on Catalyst Model Preparation, related to Results (Strategy and Catalyst Model Preparation)**

Support oxide. Doping La_2O_3 into CeO_2 in $La_{0.5}Ce_{0.5}O_{1.75}$ results in the formation of oxygen vacancies $V_O^{\bullet\bullet}$ in the oxygen sublattice. This can be represented by the following defect reaction using Kröger-Vink notation:

$$O_O^x + 2Ce_{Ce}^x + La_2O_3 \rightarrow\ 2La'_{Ce} + V_O^{\bullet\bullet} + 2CeO_2,$$

where the La^{3+} ion at the Ce lattice site La'_{Ce} has an effective charge of -1.[1] From this reaction, the condition for maintaining electrical neutrality is $2[La'_{Ce}] = [V_O^{\bullet\bullet}]$. This means that the substitution of one Ce^{4+} cation by two La^{3+} cations within the fluorite structure will result in the formation of one oxygen vacancy.

In a reducing H_2 atmosphere, as utilized in the experimental work by Ogura et al.[2], reduction at high temperatures occurs according to the following reaction[3]:

$$La_{0.5}Ce_{0.5}O_{1.75} + 0.5xH_2 \rightarrow La_{0.5}Ce_{0.5}O_{1.75-x} + 0.5xH_2O$$

Energy electron loss (EEL) spectroscopy measurements indicated that a significant fraction of Ce^{4+} near the surface of the Ru nanoparticles was reduced to Ce^{3+} at 650 °C.[2] Based on this finding, we assumed that the reduction primarily occurred in the topmost cationic layer. This aligns with reported experimental observations showing that vacancy formation predominantly occurs at the oxygen surface and subsurface, even in highly reduced states of CeO_2.[4] The definition of the reduction degree *x* used to describe the catalysts' models in the article is defined by the composition in the finite 4 x 4 x 3 slab model and does not necessarily correspond to the real system's *x* value. This discrepancy can be understood by discussing two limiting cases. For instance, the slab models for a non-reduced surface result in a composition of $La_{0.5}Ce_{0.5}O_{1.75}$. Compositions with low oxygen concentrations arise from the removal of surface oxygen atoms, with the highest removal ($x = 0.32$) observed for the Ce cationic surface due to oxygen concentration around the Ce^{4+} cations after molecular dynamics equilibration. This value of *x* is higher than the maximal possible value for the real system ($x = 0.25$), a discrepancy arising from the finite size of the slab. Adding more layers would result in lower *x* values. The main goal of this quantity is to serve as a descriptor for mapping the catalyst models (Fig. 1d in the article) and to ensure the reproducibility of our approach. Regardless, only configurations with $x \leq 0.25$ were used in the sections 'Catalyst Configuration of a Real System' and 'Analysis of Catalytic Activity' of the article to ensure a realistic degree of reduction, as these sections address real systems.

Nanoparticle configuration. We limited our study to ruthenium nanoparticles with a hexagonal close-packed (hcp) structure. This decision was made because face-centered cubic (fcc) ruthenium nanoparticles are experimentally present only when specific synthesis techniques designed for this purpose are applied[5,6], which is not the case in the experimental work[2] on which our study relies. Moreover, choosing nanoparticle configurations further requires the investigation of representative nanoparticle sizes. Comparing model nanoparticle sizes with measured sizes from real nanoparticles is not trivial. There are two types of measurements for particle sizes in general: equivalent diameters and statistical lengths[7] (see Methods S3 for definitions and calculation methods). Statistical lengths are mostly used in image evaluation methods with flat particle projection in random orientations. Ogura et al.[2] used high-angle annular dark-field scanning transmission electron microscopy (HAADF-STEM) to measure the ruthenium particle size distribution. For that reason, we found the Feret diameter to be a suitable measure for comparing particle size with the experimental particle size distribution. It is defined as the distance between two parallel lines that are tangent to the particle's profile and

perpendicular to the projection direction (see Fig. S7). Various nanoparticle configurations with various sizes were optimized (see Fig. S8). For each nanoparticle, the average and maximum Feret diameter values (d_F and $d_{F,max}$, respectively) were calculated using the projection direction provided in Fig. S8. Additionally, the diameter of the surface-equivalent sphere (d_S), the diameter of the projection area-equivalent circle (d_P), and the height (h) of the nanoparticle were also calculated for comparison.

Table S2 includes all these values for various nanoparticle configurations. This study aims to reproduce experimental data for catalyst configurations reduced at 500 °C and 650 °C. The experimental particle size distributions are given in Fig. S9, with average particle sizes of 1.8 nm and 1.7 nm, respectively. When compared to the average Feret diameter values, the Ru_{238} and Ru_{252} configurations emerge as suitable candidates for use as nanoparticle base models in this study. However, the real scenario still consists of particles of various sizes, which together form the size distribution.

To identify models that together provide a representative picture of the real size distribution, we modelled and optimized all on-top N_2 adsorption configurations for all nanoparticles. We further calculated the values for the adsorption energy (ε_{ads}), the charge of the N_2 molecule (q_{N_2}), and the N_2 wavenumber $\tilde{\nu}_{N_2}$. Using all values for each N_2 adsorption property, we created a weighted histogram, where the weighting factor depends on the experimental distribution and the nanoparticle size. This approach allowed us to calculate average histograms, representing the distribution for each adsorption property based on the experimental particle size distributions (see Methods S3). Fig. S10, Fig. S11 and Fig. S12 present histograms for each N_2 adsorption property for all 11 nanoparticle configurations. Additionally, the average histograms are overlaid on the histogram of the Ru_{238} nanoparticle in a separate diagram in each figure. These depictions make it evident that the results obtained using the Ru_{238} configuration are sufficient to reproduce a distribution similar to the average histograms for both reduction temperatures, and thus transferable to other nanoparticle sizes within the experimental distributions. Therefore, we selected this configuration. To place it on the support model, we adjusted it by cutting it at its largest projection area to maintain the same Feret diameter, resulting in the Ru_{143} model.

Nanoparticle Encapsulation. The nanoparticle encapsulation model consisted of La, Ce, and O atoms decorating the lower layers of the nanoparticle. Energy dispersive X-ray (EDX) mapping and energy EEL spectra detected Ru peaks exclusively in the upper layers of the nanoparticle (see Fig. S13).[2] In contrast, the lower layers of the Ru nanoparticles showed peaks for Ru, La^{3+}, Ce^{3+}, and Ce^{4+}, while the support material displayed peaks corresponding only to its constituent elements. These findings supported our decision to include La and Ce cations as part of the SMSI.

Ideally, the models would reconstruct atomic-resolution experimental images, enabling a detailed analysis of the structural and chemical nature of the decoration. Furthermore, creating "true digital twins" remains a significant challenge, even for simpler catalyst systems[8]. Moreover, interpreting the available HAADF-STEM images of the studied catalyst, including the one shown in Fig. S13a, still requires the application of complex approaches such as AI-based methods[9]. As an alternative, we adopted a statistical approach, where each identified set of configurations delivers average values that align with the experimental data.

Throughout this study, we demonstrate that the chosen modelling approach was sufficient to effectively handle the system, while further analysis could explore a wider range of particle sizes, oxide reduction assessed through a thermodynamic approach, and additional defects in the metal-oxide composite, modelled as a slab. Furthermore, the formation of the SMSI could be also calculated using molecular dynamics simulations.[10] Nevertheless, modelling SMSI by MD simulations was only proven for temperatures higher than 2000 °C, and thus, achieving the 328 models required to build a similar

map as proposed here would demand a substantial amount of computational time, given the much lower reduction temperatures.

DataS2: Simulated Spectra for Each Configuration, related to Fig. 3a

The dipole moment was not calculated by the Universal Neural Network Potential (UNNP) version used in the article and, thus, it was not considered in the wavenumber calculation. We repeated the calculations for the wavenumber spectra by accounting for the ^{15}N isotope. Thus, we adjusted the N mass to 15 u and compared the results with the experimental data. The spectra depicted in Fig. S16 correspond to that shown in Fig. 3a in the article. We reproduced the red-shift of the peaks of ^{14}N relative to the peaks of ^{15}N as a factor of $(m(^{14}\mathrm{N})/m(^{15}\mathrm{N}))^{1/2}$, as physically expected and experimentally confirmed. All spectra are provided in the 'Supplementary Data A' file (directory paths: ^{14}N: Figures/Catalytic/m14; ^{15}N: Figures/Catalytic/m15). The values of x, $\bar{q}_{\mathrm{Ru},0}$, and $\bar{q}_{\mathrm{N}_2}$ are summarised in the 'FreqHist_Legende_T25C-p6kPa.xlsx' file within each directory.

DataS3: Similarity Analysis of the Simulated and Measured Spectra, related to Fig. 3b

We first pre-processed the measured spectra following the literature[11]. The two measured spectra were normalised with the maximum intensity for T_{Red} = 500 °C and baseline corrected. The baseline was defined by connecting all points not included in the areas of interest. The areas of interest were defined by the points with a derivative value higher than a threshold of 0.006 plus three adjacent data points[11]. The results of this pre-processing are shown in Fig. S17.

We searched for a suitable hit quality index (HQI) to compare the calculated wavenumber distribution and the measured spectra by considering the following possibilities[11]:

$$\mathrm{HQI_{PCC}} = \frac{\sum_{i=1}^{n}(s_i - \bar{s})(r_i - \bar{r})}{\sqrt{\sum_{i=1}^{n}(s_i - \bar{s})^2 \cdot \sum_{i=1}^{n}(r_i - \bar{r})^2}}$$

$$\mathrm{HQI_{ED}} = \left(1 + \sqrt{\sum_{i=1}^{n} |s_i - r_i|^2}\right)^{-1}$$

$$\mathrm{HQI_{RMSD}} = \left(1 + \sqrt{\frac{1}{n}\sum_{i=1}^{n} |s_i - r_i|^2}\right)^{-1}$$

$$\mathrm{HQI_{JD}} = \left(1 + \sum_{i=1}^{n} s_i \ln\left(\frac{s_i}{r_i}\right) + r_i \ln\left(\frac{r_i}{s_i}\right)\right)^{-1}$$

where s_i and r_i are the intensities at specific wavenumbers for the simulated and measured spectra, respectively. $\mathrm{HQI_{PCC}}$ is the Pearson correlation coefficient, $\mathrm{HQI_{ED}}$ is based on the Euclidean distance, $\mathrm{HQI_{RMSD}}$ on the root-mean-square deviation, and $\mathrm{HQI_{JD}}$ on the Jeffrey divergence. We first performed

a cross-check by comparing both experimental spectra. Table S3 shows the results of a cross-check between the baseline-corrected spectra (see case A & Fig. S17) and between the independently normalised spectra (see case B & Fig. S18). Furthermore, we calculated the HQIs for each case considering the whole spectra, or only the regions with higher (hi: $\tilde{\nu}_{N_2}$ ≥ 2050 cm^{-1}) or lower wavenumbers (lo: $\tilde{\nu}_{N_2}$ < 2050 cm^{-1}). A value higher than 0.60 indicates that the spectra are similar[11]. While the values for the HQI_{PCC} and the HQI_{RMSD} suggested that both experimental spectra are highly similar, the HQI_{JD} values suggested no similarity, even for the cases where a high similarity was expected, such as $\mathrm{HQI}_{\mathrm{JD,B}}^{\mathrm{hi}}$ (see Table S3 & Fig. S18). Moreover, it was possible to differentiate the baseline-corrected spectra of case A using HQI_{ED}, resulting in $HQI_{ED,A} = 0.23$. However, this differentiation occurs mainly to the disparity in the high-wavenumber region, because $\mathrm{HQI}_{\mathrm{ED,A}}^{\mathrm{lo}}$= 0.70 suggests that the low-wavenumber region is similar for both spectra. This trend was opposite for the independently normalised spectra (case B), where the non-similarity of the whole spectra, $HQI_{ED,B} = 0.37$, resulted from the differentiation of the low-wavenumber region ($\mathrm{HQI}_{\mathrm{ED,B}}^{\mathrm{lo}}$ = 0.39) by showing a high similarity at the high-wavenumber region ($\mathrm{HQI}_{\mathrm{ED,B}}^{\mathrm{hi}}$ = 0.63). This result is consistent with Fig. S18.

We used $HQI_{ED,B}$ to compare the simulated wavenumber distribution and measured spectra. The results are depicted in Fig. S19 as a function of the reduction degree *x*, where only configurations with *x* ≤ 0.25 where considered. Furthermore, Fig. S20 shows an example of the calculated normalised wavenumber distribution for all configurations corresponding to one SS cationic surface categorised by *x*. For no surface reduction, the wavenumber distribution within the low-wavenumber region is not as pronounced as for higher *x*. This means that the spectra should be more similar to each other and thus, have different HQI values compared to each of the experimental spectra. This behaviour is well reproduced by the values for $HQI_{ED,B}$ in Fig. S19. As *x* increases, the values for $HQI_{ED,B}$ overlap significantly due to the high values of $\mathrm{HQI}_{\mathrm{ED,B}}^{\mathrm{hi}}$, which also show a high value of 0.63 between both experimental spectra (see Table S3). Nevertheless, differentiation is possible when the values for $\mathrm{HQI}_{\mathrm{ED,B}}^{\mathrm{lo}}$ are used. Therefore, it is suitable to limit the further comparison to the low-wavenumber region. For simplicity, we refer to $\mathrm{HQI}_{\mathrm{ED,B}}^{\mathrm{lo}}$ as $\mathrm{HQI}_{\mathrm{B}}^{\mathrm{lo}}$ hereafter.

Next, we calculated the cumulative averages of the wavenumber distribution by averaging the configurations sorted in descending order of their $\mathrm{HQI}_{\mathrm{B}}^{\mathrm{lo}}$ value compared to the experimental spectra. The first 10 cumulative averages are depicted in Fig. S21. The lowest blue spectrum corresponds to the configuration with the highest HQI_B(T_{Red} = 500 °C), followed by the spectrum corresponding to the average of the two configurations with the highest $\mathrm{HQI}_{\mathrm{B}}^{\mathrm{lo}}$($T_{Red}$ = 500 °C) values, and so on. The samples obeying the same rule but similar to the experimental IR spectra for catalysts reduced at 650°C are also provided in Fig. S21 for comparison. We then calculated the $\mathrm{HQI}_{\mathrm{B}}^{\mathrm{lo}}$ value of the average spectrum $\mathrm{HQI}_{\mathrm{B,Ave}}^{\mathrm{lo}}$ (Fig. S22). High values for $\mathrm{HQI}_{\mathrm{B,Ave}}^{\mathrm{lo}}$ were achieved after averaging for both cases. The progression of $\mathrm{HQI}_{\mathrm{B,Ave}}^{\mathrm{lo}}$($T_{Red}$ = 500 °C) (blue markers) showed a plateau, making it unclear which average provides the best match. Fig. S22 also shows the low-wavenumber peak $\tilde{\nu}_{\mathrm{N_2,Ave}}^{\mathrm{lo}}$ for the cumulative averages. Following the well-known method of peak matching[11], we chose the configuration combination with the nearest peak to the experimental value (Table S4). We then disregarded all configurations with Ce and La cationic surfaces to ensure consistency with the oxide description in Data S1. The homogeneity of the SS state is discussed in more detail in Data S4. This resulted in 40 configurations that were used to calculate the final average for T_{Red} = 500 °C depicted in Fig. 3b of the main article.

We could not apply the same methodology to find a match for T_{Red} = 650 °C, because even the configuration combination with the highest $\mathrm{HQI}_{\mathrm{B,Ave}}^{\mathrm{lo}}$($T_{Red}$ = 650 °C) value showed a peak far from the

expected experimental value of 1844 cm^{-1}. As a second attempt, we repeated the cumulative averaging procedure by averaging the configurations sorted in ascending order of the difference $\Delta\tilde{\nu}^{\mathrm{lo}}_{\mathrm{N_2}} = |\tilde{\nu}^{\mathrm{lo}}_{\mathrm{N_2}} - \tilde{\nu}^{\mathrm{lo}}_{\mathrm{N_2,Exp}}|$. The results for the $\mathrm{HQI}^{\mathrm{lo}}_{\mathrm{B,Ave}}$ values and the resulting peaks $\tilde{\nu}^{\mathrm{lo}}_{\mathrm{N_2,Ave}}$ are given in Fig. S23. This procedure resulted in an outstanding match between the simulated and measured peaks. However, the $\mathrm{HQI}^{\mathrm{lo}}_{\mathrm{B,Ave}}$ values are not as high as those in Fig. S22.

From these results, the optimisation problem becomes finding the combination that shows the highest $\mathrm{HQI}^{\mathrm{lo}}_{\mathrm{B,Ave}}$($T_{\mathrm{Red}}$ = 650 °C) value and is also an acceptable match for the peak. Among other possibilities, we used a genetic algorithm (GA) to solve this problem (see details in the Methods S4). We considered only the 28 configurations with the highest $\mathrm{HQI}^{\mathrm{lo}}_{\mathrm{B,Ave}}$($T_{\mathrm{Red}}$ = 650 °C) values (Fig. S24) because these configurations showed values above the highest value for $\mathrm{HQI}^{\mathrm{lo}}_{\mathrm{B,Ave}}$ (T_{Red} = 650 °C) within the configurations chosen to build the final average for T_{Red} = 500 °C (vertical dashed line).

We used $\mathrm{HQI}^{\mathrm{lo}}_{\tilde{\nu},\mathrm{Ave}}$ as a fitness factor if $\Delta\tilde{\nu}^{\mathrm{lo}}_{\mathrm{N_2}} \geq 3\ \mathrm{cm}^{-1}$ and the following $\mathrm{HQI_{GA}}$, based on a combination of HQIs as a fitness factor otherwise:

$$\mathrm{HQI_{GA}} = \mathrm{HQI}^{\mathrm{lo}}_{\mathrm{B,Ave}} \cdot \mathrm{HQI}^{\mathrm{lo}}_{\tilde{\nu},\mathrm{Ave}}$$

where $\mathrm{HQI}^{\mathrm{lo}}_{\tilde{\nu}}$ is the HQI used to describe the difference $\Delta\tilde{\nu}^{\mathrm{lo}}_{\mathrm{N_2}}$. As the $\mathrm{HQI_{PCC}}$ and $\mathrm{HQI_{RMSD}}$ are designed to consider more than one value, using the $\mathrm{HQI_{ED}}$ or $\mathrm{HQI_{JD}}$ may be more suitable to describe $\Delta\tilde{\nu}^{\mathrm{lo}}_{\mathrm{N_2}}$. The optimisation may focus on a high $\mathrm{HQI}^{\mathrm{lo}}_{\mathrm{B,Ave}}$ value by allowing some discrepancies regarding the peak position, as such discrepancies are generally acceptable[11]. Therefore, $\mathrm{HQI}^{\mathrm{lo}}_{\tilde{\nu}}$ should not be strongly affected by small $\Delta\tilde{\nu}^{\mathrm{lo}}_{\mathrm{N_2}}$. Fig. S25 shows the progression of the $\mathrm{HQI}^{\mathrm{lo}}_{\tilde{\nu}}$ based on the ED and JD as functions of the difference $\Delta\tilde{\nu}^{\mathrm{lo}}_{\mathrm{N_2}}$. The $\mathrm{HQI}^{\mathrm{lo}}_{\tilde{\nu}}$ based on the ED strongly decreases reaching a value of 0.1 for a change of 5 cm^{-1}. In contrast, the $\mathrm{HQI}^{\mathrm{lo}}_{\tilde{\nu}}$ based on the JD decreases slowly for small values reaching a value of around 0.94 for a change of 10 cm^{-1}. Nevertheless, it decreases much faster for high differences. Therefore, $\mathrm{HQI}^{\mathrm{lo}}_{\tilde{\nu}}$ based on JD was chosen to describe $\Delta\tilde{\nu}^{\mathrm{lo}}_{\mathrm{N_2}}$.

The results for the first 500 iterations from the optimisation using the GA are provided in Fig. S28. After 450 iterations, a solution was found that remained unchanged for the remaining iterations. The configuration set consisted on 7 of the 28 initial configurations resulting in a high $\mathrm{HQI}^{\mathrm{lo}}_{\mathrm{B,Ave}}$ value (>0.60) and effectively reproducing the peak of the experimental IR. The resulting average for T_{Red} = 650 °C is the one provided in Fig. 3 of the main article. Note that the simple bottom-up approach shows a certain discrepancy with the measured broadening, which may originate from the overestimation of the considered broadening mechanisms and other effects associated with the measurement that are not included in our calculations. All results for the simulated distributions can be found in the 'FreqHist_T25C_p6kPa.xlsx' file within the 'Doc' directory of the 'Supplementary Data A' file.

Data S4: Catalyst Configurations Reproducing Experimental Spectra, related to Results Fig. 3b

The set of catalyst configurations corresponding to T_{Red} = 500 °C and 650 °C are provided in Fig. S26. Various average properties for these sets are summarized in Table S5. Wigner-Seitz (WS) defect analysis[12] was used to calculate the number of vacancies on the cationic surface (Methods S5). The absolute average values for the reduction degree *x* and the average charge $q_{\mathrm{Ru,0}}$ of the Ru atoms on the nanoparticle surface layer are larger for T_{Red} = 650 °C, though they do not exceed twice the values for T_{Red} = 500 °C. Additionally, the average number of oxygen vacancies $N_{\mathrm{V_O^{\bullet\bullet}(S)}}$ on the cationic surface (S) appears to be the similar for both temperatures, despite the differing reduction degrees.

By modelling the artificial SMSI, La, Ce and O atoms were transferred from the cationic surface to the nanoparticle, creating cation vacancies and additional oxygen vacancies at the surface. Additionally, surface reconstruction can displace cations from their original lattice sites, leading to local atomic arrangements that deviate from the fluorite structure (see Fig. S27). In both scenarios, the oxygen atoms surrounding the cation vacancies or displaced cations underwent rearrangement during optimization. These oxygen atoms migrated to subsurface sites, became part of the nanoparticle encapsulation layers, or contributed to new local arrangements within the cationic surface formed by displaced La and Ce atoms. This kind of surface reconstruction resembles the formation of Schottky defects in behaviour[13]. $N_{V_O^{\bullet\bullet}(S)}$ was corrected accordingly (see Methods S5), resulting in a higher value for T_{Red} = 500°°C. However, the proportion of $N_{V_O^{\bullet\bullet}(S)}$ to the number of cations on the cationic surface $N_{Ce(S)+La(S)}$ do not differ substantially, being 1.62 for T_{Red} = 500°°C and 1.53 for T_{Red} = 650°°C. In terms of the number of oxygen atoms, the resulting proportions to the number of cations on the cationic surface are 1.29 and 1.56, respectively.

In multinary materials, cation disorder refers to the deviation of cation atomic positions from a regular crystalline structure within the cationic sublattice.[14] The analysis of cation disorder in transition metal oxides involves evaluating the displacement of cations from the crystalline structure[15] and their short-range order[16]. From the WS defect analysis (see Methods S5), the SMSI and the cationic surface reconstruction were identified as the main reasons for the displacement of Ce, La, and O atoms (see Fig. S27). The mean values of the neighbour distances $r_{i\text{-}j}$ (i,j=Ce,La) over the configurations for each reduction temperature are provided in Table S6 to confirm any other substantial displacement within the atoms of the slab's cationic sublattice. All the values are equal within statistical uncertainty, indicating that there are no substantially displaced cations beyond those already identified.

Regarding the number of displaced atoms ($N_{La(D)}$, $N_{Ce(D)}$ and $N_{O(D)}$ in Table S5), $N_{La(D)}$ is statistically equivalent to $N_{Ce(D)}$ for both T_{Red} = 500°°C and T_{Red} = 650°°C. Furthermore, $N_{Ce(D)}$ and $N_{La(D)}$ are approximately twice as high, and $N_{O(D)}$ remains similar for T_{Red} = 650°°C compared to T_{Red} = 500°°C. The proportions of the number of oxygen atoms to the displaced La and Ce atoms are 0.5 and 0.26 for T_{Red} = 500°°C and T_{Red} = 650°°C, respectively. Our configurational map captures a broad range of defect numbers for each reduction degree. Nevertheless, our identification method resulted in configurations for T_{Red} = 500 °C, that, on average, exhibit fewer defects than those for T_{Red} = 650 °C. This is consistent with the calculated energetics, where a higher reduction degree may lead to a greater number of defects (see Methods S5).

The homogeneity of the SS state of the cationic sublattice was evaluated using the Warren-Cowley[17] parameter α_{ij} (see Methods S5). Values close to zero indicate a random mixing of La and Ce in the cationic sublattice. As shown in Table S6, all values are close to zero, indicating a random mixing and no substantial difference in the short-range order of the configurations when comparing both catalyst reduction temperatures. On one hand, since no further GA optimization was performed for T_{Red} = 500, we manually disregarded the configurations with Ce and La, as mentioned in Data S3. We kept the configurations with superlattice (SL) cationic surfaces because the Warren-Cowley parameters for those configurations were similar to those of the SS configurations. On the other hand, the GA optimization for T_{Red} = 650°°C converged toward SS cationic surface configurations, where only one of the cationic surfaces exhibited an SL configuration, and no slab configurations with Ce or La cationic surfaces were identified, in accordance with the oxide description provided in Data S1.

This discussion lacks an analysis of polarons within the oxide configurations. Unfortunately, the electronic structure cannot be calculated using the UNNP, preventing further analysis of this important feature, which remains an open issue. The discussed average values, along with the individual values

for all 200 configurations, can be found in the ‘FreqHist_T25C_p6kPa.xlsx’ file in the ‘Doc’ directory of the ‘Supplementary Data A’ file.

Data S5. Structural Analysis of Adsorption States, related to Fig. 3d

To analyse the structures of the adsorption states (Fig. 3d in the article), we first searched for variables to determine the local structure of the adsorption state. We used the centre of mass of the N_2 molecule, defined a cut-off radius, and counted the number of cations $N_{\mathrm{Ce+La}}$, the number of displaced cations $N_{\mathrm{Ce(D)+La(D)}}$, the number of oxygen atoms N_{O} and the number of oxygen vacancies $N_{\mathrm{V}_{\mathrm{O}}^{\bullet\bullet}}$ for the adsorption states given in Fig. 3d. The results for different cut-off radii are summarised in Table S7.

In states A to D, cations around the adsorbed molecules first appear at a distance of 4 Å, while in states E to I, they appear at 3 Å. This indicates that the closer the adsorption state is to a Ce or La cation, the lower the N_2 wavenumber. This observation may also explain the higher activation barrier for states A, B, and D compared to the other states, although it does not clarify the similar barriers between states C, E, G, and H. Notably, the primary difference lies in the proximity to the oxide surface for states C and E. Adsorption states close to the surface, such as C and E, demonstrate similar activity levels to those more distant from the surface but surrounded by displaced atoms encapsulating the nanoparticle, like states G and H. A combination of these effects can occur through local arrangements of oxidized Ce or La atoms, akin to Schottky defects, which encapsulate the bottom layer of the nanoparticle and extend the support oxide, along with Ce or La atoms in the upper nanoparticle layers (refer to Fig. S27 for an example catalyst configuration with such a combination of features). This combination of features characterizes states F and I. Lastly, no active role of the oxygen vacancies could be deduced in influencing the catalytic activity. Using a cut-off radius of 5 Å or higher, we defined the following conditions for counting the number of adsorption states in each configuration:

$$\mathrm{A}: \tilde{\nu}_{\mathrm{N}_2} \geq 2100\ \mathrm{cm}^{-1}, N_{\mathrm{Ce+La}} = N_{\mathrm{O}} = 0$$

$$\mathrm{B,D}: 2100\ \mathrm{cm}^{-1} \geq \tilde{\nu}_{\mathrm{N}_2} \geq 1900\ \mathrm{cm}^{-1}, N_{\mathrm{Ce+La}} > 0, N_{\mathrm{O}} > 0, \Delta z > 0.5\ \text{Å}$$

$$\mathrm{C,E}: 2100\ \mathrm{cm}^{-1} \geq \tilde{\nu}_{\mathrm{N}_2} \geq 1900\ \mathrm{cm}^{-1}, N_{\mathrm{Ce+La}} > 0, N_{\mathrm{O}} > 0, \Delta z < 0.5\ \text{Å}$$

$$\mathrm{G,H}: 1800 < \tilde{\nu}_{\mathrm{N}_2} \leq 1900\ \mathrm{cm}^{-1}, N_{\mathrm{Ce+La}} > 0, N_{\mathrm{O}} = 0$$

$$\mathrm{F,I}: 1700 < \tilde{\nu}_{\mathrm{N}_2} \leq 1900\ \mathrm{cm}^{-1}, N_{\mathrm{Ce+La}} > N_{\mathrm{O}}, N_{\mathrm{O}} > 0$$

where Δz denotes the distance to the surface in the *z* direction. We demonstrate the effectiveness of this categorisation in the main manuscript.

Data S6: Further Discussion on Catalytic Activity, related to Results (Analysis of Catalytic Activity)

From Data S5, we can summarize that the main effect for higher catalytic activity in states F and I results from the combination of two features: Ce or La atoms at the nanoparticle and a Schottky-like defect structure surrounding the nanoparticle at the bottom. Both features are primarily due to cationic disorder (in terms of Ce and La cation displacement) resulting from the SMSI and surface reconstruction. Therefore, cationic disorder due to displacement can be understood as the most crucial mechanism behind the creation of highly active sites. This is consistent with the discussion in Data S4, which compares in detail the configuration sets for T_{Red} = 500 °C and T_{Red} = 650 °C. The primary difference between these sets lies in the number of displaced Ce, La, and O atoms. High active sites are not unique to configurations with T_{Red} = 650 °C. Configurations for both cases can exhibit such high

active sites. Therefore, counting the number of appearances is a suitable way to differentiate, as done in the article.

Finally, the resulting unique geometry, which enables the sandwiching of N_2 molecules, is unsurprisingly the most active configuration. This arrangement effectively combines both features, placing the adsorption configuration in the free space between single cations at the nanoparticle and the Schottky-like defect structure.

Data S7, Supplemental Data Descrption, related to Data and code Availability

Depictions of different views of all 25 initial configurations of the catalyst with a solid-solution (SS) cationic surface, their optimised structures, and the resulting charge distribution can be found in the 'Supplementary Data A' file (directory paths: Figures/Catalyst/Initial; Figures/Catalyst/Initial/Optimization). The depictions of other configurations may be obtained from the authors upon reasonable request. The optimised Ru_{238} HCP nanoparticle structure and frames for the configurations labelled 165-La-1-1-xO25-1Cat75 and 165-La-1-1-xO25-1CatO75 are given in Fig. S14. The directories containing the figures are labelled in the following manner. The number '165' refers to the rotational angle β of the nanoparticle relative to the optimised structure and is followed by the cationic surface composition of the support (here, 'La'). The first number in '1-1' refers to the configuration number, and the second refers to the number of reduced cationic layers. 'xO25' refers to the percentage of oxygen remaining in the layers after reduction (e.g. '25' corresponds to a 75% reduction in oxygen). The numbers '1' and '75' in '1Cat75' refer to the number of nanoparticle layers wrapped by cations and percentage of occupied Ru atoms at the lowest nanoparticle layer, respectively. If oxygen atoms were relocated to the nanoparticle decoration, the directory was labelled as 'CatO'. All values are relative to the initial configuration. For reproducibility and following the described nomenclature, the XYZ files for all initial and final configurations are provided in the 'Supplementary Data B' file.

Methods S1: Energetic Analysis of the Ru_{143} Half-Nanoparticle Orientation on the Support, related to METHOD DETAILS (Model Preparation and Calculation)

The nanoparticle is rotated by an angle of β = 165° for the 200 models used in the study. The calculated values of the interfacial energy ε_{inter} between the nanoparticle and support for 20 catalyst models and 5 nanoparticle orientations are summarised in Table S8. The chosen angular range was sufficiently wide to achieve accurate analysis owing to the symmetry of the support. The interfacial energy was calculated as follows:

$$\varepsilon_{\mathrm{inter}} = \varepsilon_{\mathrm{Ru238-La_{0.5}Ce_{0.5}O_{1.75}}} - \frac{N_{\mathrm{Ru_{143}}}}{N_{\mathrm{Ru_{238}}}}\varepsilon_{\mathrm{Ru238}} - \varepsilon_{\mathrm{La_{0.5}Ce_{0.5}O_{1.75}}}$$

where $\varepsilon_{\mathrm{Ru238-La_{0.5}Ce_{0.5}O_{1.75}}}$ is the potential energy of the optimised catalyst structure with a non-reduced support, *N* is the number of atoms for the (half) Ru nanoparticle, and $\varepsilon_{\mathrm{La_{0.5}Ce_{0.5}O_{1.75}}}$ is the potential energy of the non-reduced slab after the MD simulation. In general, the nanoparticle rotated by β = 165° is the most energetically favourable. The orientation for this particular case can be taken from the top view of Fig. S14.

Methods S2: Validation of the UNNP for Describing Ru Nanoparticles, La-Ce-Ru-O Interactions, and N_2 Adsorption on Ru, related to METHOD DETAILS (Simulation Methods)

We validated the UNNP for describing Ru nanoparticles in a previous work[18] by calculating the cohesive energy of Ru nanoparticles with various structures and sizes[19]. The results for the hcp nanoparticles are reproduced in Fig. S15. The linear regression analysis comparing the UNNP and density functional theory (DFT) values for the hcp structure resulted in R^2 values of 0.999, with slopes of 6.08(10) and 5.913(97), respectively. These statistically equivalent slopes demonstrate the accuracy of UNNP in describing Ru hcp nanoparticles. Notably, the absolute deviation between the UNNP and DFT values for the Ru_{238} nanoparticle was only 0.074 eV.

Various bulk systems containing La, Ce, Ru, and O were calculated using structures from the Materials Project Database[20] to further validate the accuracy of the UNNP. While only one configuration was available for La-Ce-Ru and La-Ce-O, we selected only experimentally observed configurations for all other combinations. The results are summarized in Table S9 and Table S10. The RMSD values for the cell lengths *a*, *b* and *c* were 0.31 Å, 0.28 Å, and 0.18 Å, respectively, and for the cell angles α, β, and γ, 0.51°, 1.7°, and 2.7°, respectively, demonstrating the accuracy of the UNNP in describing the interactions between these elements. The optimised $La_2Ce_2O_7$ configuration with a cubic crystal system corresponds to a $La_{0.5}Ce_{0.5}O_{1.75}$ configuration with a fluorite structure. The calculated lattice constant of 7.980 Å deviates from the DFT value of 7.939 Å by only 0.52%. Additionally, the calculated bulk modulus (131 GPa) is in good agreement with the value recorded in the Materials Project Database (130 GPa).

To validate the accuracy of the UNNP for calculating the adsorption of N_2 on Ru, we summarised all property values calculated using the UNNP or DFT and experimental values obtained from previous studies (Table S11). In Table S12, we present a comparative analysis of the DFT values calculated by Rivera et al.[21] and the UNNP values for various properties of N_2 adsorption configurations on the on-top sites of a Ru_{153} nanoparticle. The RMSD for adsorption energy, wavenumber, and interatomic N-N distance were 0.122 eV, 14 cm^{-1}, and 0.001 Å, respectively. These results further confirm the accuracy of the UNNP model.

As discussed in the main article, Table S1 compares the calculated Gibbs energy barriers for the dissociation of an N_2 molecule on a Ru slab with DFT values. This comparison demonstrates that the UNNP model accurately describes this quantity.

Methods S3: Nanoparticle Size Quantification and Average Histograms, related to Results (Strategy and Catalyst Model Preparation)

The diameter of the surface-equivalent sphere (d_S) and the diameter of the projection area-equivalent circle (d_P) are given by[7]:

$$S = \pi \cdot d_S^2 \rightarrow d_S = \sqrt{\frac{S}{\pi}},$$

$$A_P = \frac{\pi}{4} d_P^2 \rightarrow d_P = \sqrt{\frac{4 \cdot A_p}{\pi}},$$

where S is the surface are of the nanoparticle and A_P the projection area defined. The definition of the height *h* of the nanoparticle is given in Fig. S8. The definition of the Feret diameter $d_{F,i}$ for a specified measurement direction *i* is depicted in Fig. S7. The average Feret diameter d_F is the mean value over all calculated $d_{F,i}$ values, while $d_{F,max}$ is the maximum among them. The positions of atoms in the relaxed nanoparticle configurations were used to roughly approximate all the geometrical quantities described in this section.

We used the calculated values for a specific N_2 adsorption property *x* ($x = \varepsilon_{ads}$, q_{N_2}, or $\tilde{\nu}_{N_2}$) among all nanoparticle configurations to construct the average histograms by counting the number of absolute frequencies for each bin as follows:

$$N_{\mathrm{bin}} = \sum_{\forall x \in \mathrm{bin}} \frac{w(T_{\mathrm{Red}}, d_{\mathrm{F}})}{N_{\mathrm{NP}}(d_{\mathrm{F}})},$$

where *w* represents a weighting factor depending on the reduction temperature T_{Red} and the average Feret diameter d_F. We consider nanoparticles to have a similar Feret diameter if those diameters fall within the same size category. The size categories are defined by the experimental particle size distribution (see Fig. S9). Values from a nanoparticle with an average Feret diameter within a specific size category are weighted by the corresponding experimental relative frequency. The values are averaged over the number of nanoparticles N_{NP} in each category to avoid overrepresentation. Table S13 contains all numerical values used to construct the average histograms.

All histograms are constructed using 25 bins in the intervals [-1.5,0] eV for ε_{ads}, [-0.2, -0.05] e for q_{N_2} and [2200,2100] cm^{-1} for $\tilde{\nu}_{N_2}$.

Methods S4: Details of the GA, related to Fig. 3b

A flow chart of the GA is given in Fig. S31. The GA is binary and based on classical operators[22]. The number of digits for each instance is the same as the number of considered configurations for averaging the wavenumber distribution. Each digit corresponds to a configuration, where 0 and 1 mean that the configuration is taken or not taken into consideration for averaging, respectively. The initial population consists of 60 random individuals. The population undergoes classical crossover, mutation, and selection operators. The crossover is a single-point crossover swapping the half of two random chosen parent instances to create 50 new instances. Mutation is performed by creating five new

instances, each resulting from a copy of a randomly selected instance, where a randomly chosen digit was changed. The selection process is a variant of the well-known fitness proportionate selection. In each step, only the instances that have not been already selected are considered and the probabilities and cumulative probabilities are dynamically updated throughout the process. If the highest fitness value of the population remains unchanged after two iterations, a new population is created. This new population consists of the top five fittest instances, five instances selected by roulette wheel selection from the previous population, and newly generated random instances. This approach aims to facilitate convergence toward a global maximum.

We tested the algorithm to find the global maximum for the following function within the limits $-10 \leq x \leq 10$ and $-10 \leq y \leq 10$.

$$z(x, y) = 10\,(\cos(x) + \cos\,(y)) - 0.2(x^2 + y^2) + 60$$

Based on the results in Fig. S32, this function shows multiple local maxima, but only one global maximum at *x* = 0 and *y* = 0. We used instances with 30 bits (15 for *x* and 15 for *y*) to conduct the test. The optimisation results always converged to the global maximum within 100 iterations

Methods S5: Wigner-Seitz Defect Analysis, related to Fig3b

The WS defect analysis was used to identify displaced atoms and vacancies as implemented in OVITO[12]. Using the ideal fluorite structure with a cell size as large as the analysed slab configuration as a reference, the WS cell can be defined for each site in the reference cell. After comparing both configurations, atoms are assigned to a single cell. If no atom is assigned to a cell, it is considered empty, indicating a vacancy. The reference structure does not contain any vacancies; thus, the formation of vacancies from the mixing of CeO_2 and La_2O_3 is also considered. Atoms with a large displacement will leave their original WS cell and occupy another. Due to the relatively large displacements of oxygen anions compared to the ideal structure, the analysis was performed in two steps: first, calculating the cationic defects by considering only the cationic sublattice and ensuring sufficiently large WS cells for the cations, followed by the calculation of anionic defects while considering the entire system. Fig. S27 shows an example configuration with displaced Ce and La atoms coloured in green and displaced O atoms in yellow. As illustrated, the method effectively identifies displaced atoms, including those partially encapsulating the nanoparticle, those forming local structures different from the reference structure due to SMSI, and those displaced due to surface reconstruction. No displaced cations were found in slab layers other than the cationic surface. However, a few oxygen anions were displaced in lower layers. To account for this, we filtered them out by considering only neighbours of displaced cations or nanoparticle atoms.

Assuming that the surface reconstruction behaves like the formation of Schottky defects, we can describe this behaviour by the following quasi-chemical reactions[13]:

$$\mathrm{V}_{\mathrm{La}}' + 2\mathrm{V}_{\mathrm{O}}^{\bullet\bullet} \rightarrow \mathrm{nil},$$

$$\mathrm{V}_{\mathrm{Ce}}' + 2\mathrm{V}_{\mathrm{O}}^{\bullet\bullet} \rightarrow \mathrm{nil}.$$

Therefore, the number of vacancies will be overestimated as the displaced Ce and La atoms, along with the rearranged oxygen atoms, leave empty WS cells on the cationic surface. For this reason, the number of vacancies at the cationic surface is corrected by:

$$N_{\mathrm{V}_{\mathrm{O}}^{\bullet\bullet}(\mathrm{S})} = N_{\mathrm{V}_{\mathrm{O}}^{\bullet\bullet}(\mathrm{S})}^{\mathrm{WS}} - 2 \cdot \left(N_{\mathrm{Ce(D)+La}(D)}\right),$$

where WS indicates the identified vacancies from the defect analysis. Consistently, we calculate $N_{Ce(S)+La(S)}$ by counting the non-displaced cations on the cationic surface (S).

A large number of defects resembling Schottky defects is unlikely to form on slabs with the small surface area of our catalyst models. To assess the likelihood of the formation of such defects forming on supported nanoparticles, we calculated the energy differences $\Delta e_{\mathrm{D,i}}^{\mathrm{SBNP}}$ and $\Delta e_{\mathrm{D,i}}^{\mathrm{SB}}$ for each catalyst model as follows:

$$\Delta e_{\mathrm{D,i}}^{\mathrm{SBNP}} = \frac{1}{3N_{\mathrm{La(D)+Ce(D)}}}\left(E_{\mathrm{tot,i}}^{\mathrm{SBNP}} - E_{\mathrm{0,i}}^{\mathrm{SBNP}}\right),$$

$$\Delta e_{\mathrm{D,i}}^{\mathrm{SB}} = \frac{1}{3N_{\mathrm{La(D)+Ce(D)}}}\left(E_{\mathrm{D,i}}^{\mathrm{SB}} + N_{\mathrm{La(D)}}E_{\mathrm{La}} + N_{\mathrm{Ce(D)}}E_{\mathrm{Ce}} + \frac{1}{2}N_{\mathrm{O(D)}}E_{\mathrm{O_2}} - E_{\mathrm{0,i}}^{\mathrm{SB}}\right).$$

Here, $E_{\mathrm{tot,i}}^{\mathrm{SBNP}}$ denotes the total energy of the supported nanoparticle configuration *i*, while $E_{\mathrm{0,i}}^{\mathrm{SBNP}}$ represents the total energy of the supported nanoparticle without decoration. Thus, the energy difference $\Delta e_{\mathrm{D,i}}^{\mathrm{SBNP}}$ is proportional to the formation energy of the decoration atoms. Additionally, we removed the decoration and nanoparticle atoms and optimized the slab, with its energy denoted as $E_{\mathrm{D,i}}^{\mathrm{SB}}$. The energy $E_{\mathrm{0,i}}^{\mathrm{SB}}$ corresponds to the optimised slab without defects caused by nanoparticle decoration. E_{La}, E_{Ce} and $E_{\mathrm{O_2}}$ correspond to the UNNP energies of a single La atom, a single Ce atom (both computed as zero), and an oxygen molecule in vacuum (-6.02 eV), respectively. The difference $\Delta e_{\mathrm{D,i}}^{\mathrm{SB}}$ follows the definition of Hinuma *et al.*[23] for the formation energy of single defects on a pure slab. The energy differences are calculated as per-formula-unit averages based on the Schottky defect model.[24]

In Table S14, values for both energy differences are exemplarily summarized for a set of configurations with an SS cationic surface, along with the corresponding values of x and the total number of defects forming the nanoparticle encapsulation $N_D = N_{La(D)+Ce(D)+O(D)}$. It can be seen that all values for $\Delta e_{\mathrm{D,i}}^{\mathrm{SBNP}}$ are lower than those for the pure slab $\Delta e_{\mathrm{D,i}}^{\mathrm{SB}}$, indicating that the nanoparticle significantly stabilizes the formation of these defects Furthermore, higher reduction degrees correspond to the lower values of $\Delta e_{\mathrm{D,i}}^{\mathrm{SBNP}}$.

Finally, the Warren-Cowley parameter α_{ij} used to analyse the SS state is given by:

$$\alpha_{\mathrm{ij}} = 1 - \frac{p_{\mathrm{ij}}}{c_{\mathrm{j}}},$$

where p_{ij} represents the probability of finding an atom of element j surrounding an atom of element i, and c_j the overall composition of j atoms in the compound.

All values introduced in this section for all catalyst configurations are available in the 'WSAnalysis.xlsx' file, located in the Doc directory of the 'Supplementary Data A' file.

Supplemental Figures

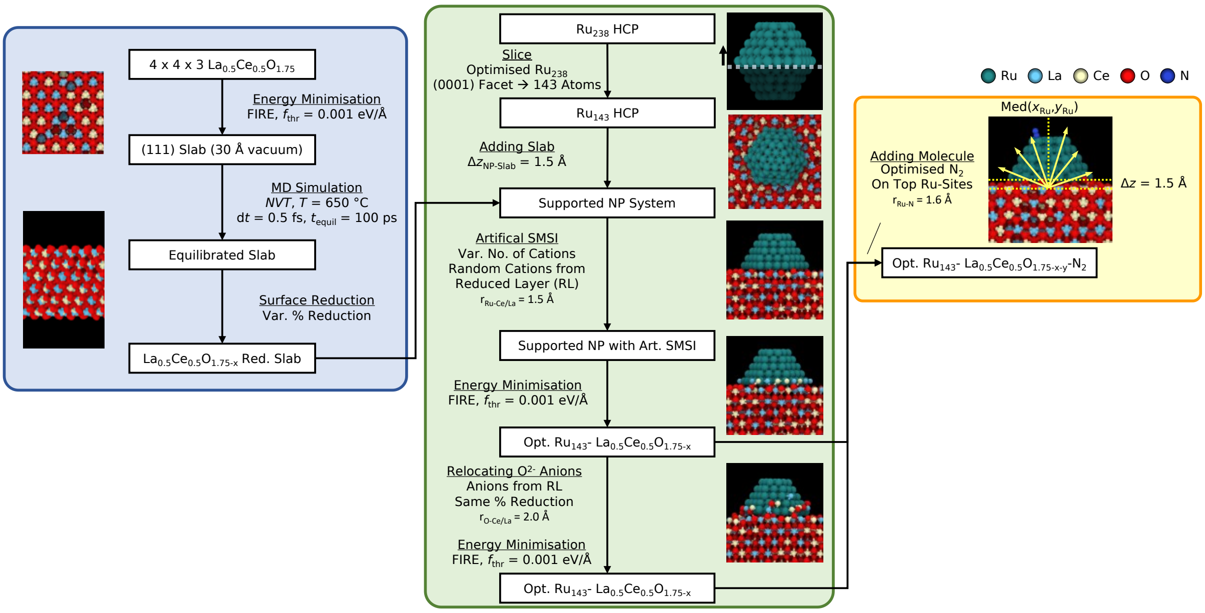

Fig. S1 Methods applied to model the catalyst systems.

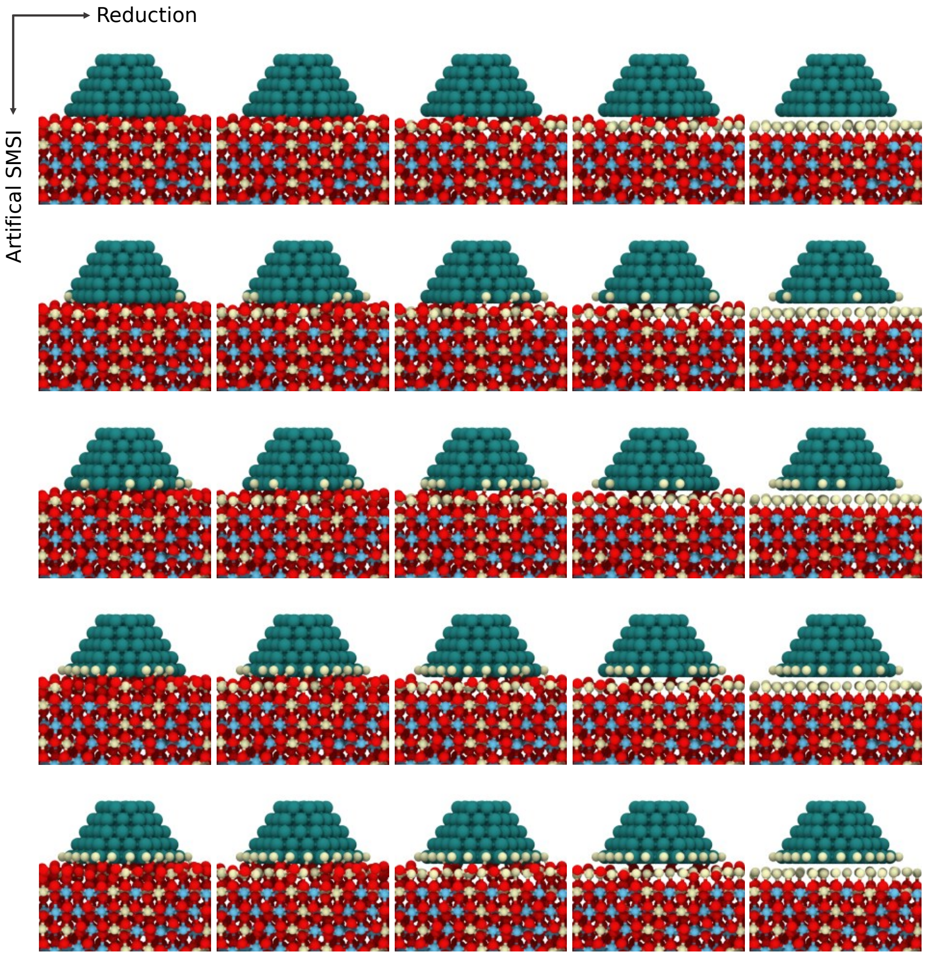

Fig. S2 Example of 25 initial configurations obtained by varying the reduction of the Ce cationic surface and artificial SMSI. The cationic surface was reduced as follows from left to right: no removal of oxygen anions, removal of 1/4 of the oxygen anions, and so on, until the complete removal of oxygen anions on the cationic surface was achieved. The artificial SMSI follows a similar rule from top to bottom, and the number of cations attached to the lowest nanoparticle layer is varied.

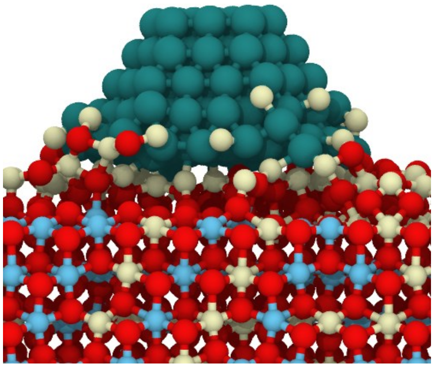

Fig. S3 Optimised structure of the configuration exhibiting high SMSI and a high reduction degree of $x = 0.24$.

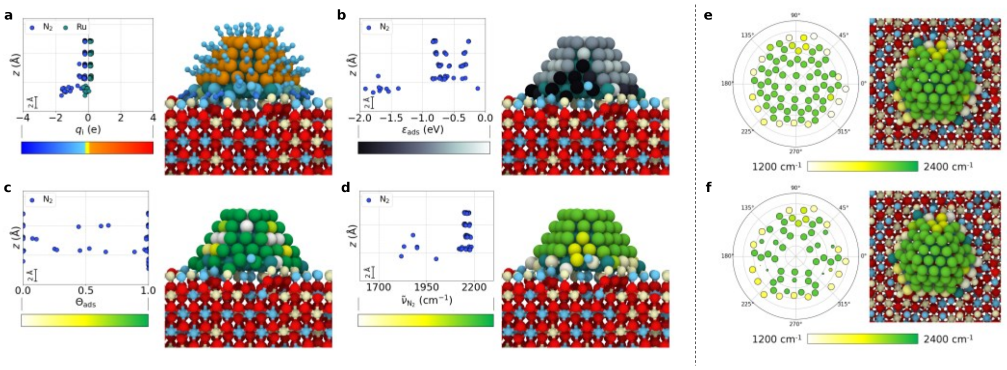

Fig. S4 a–d, Spatial maps of the (**a**) charge q_i for individual adsorbed N_2 molecules on the Ru site, (**b**) adsorption energy ε_{ads}, (**c**) N_2 coverage $\theta(T = 25\ °C, p = 6\ kPa)$, and (**d**) wavenumber $\tilde{\nu}_{N_2}$. e, f, (e) Weighted and (**f**) non-weighted spatial maps of the N_2 wavenumber in polar coordinates (weighting factor for the marker size: $\theta(T = 25\ °C, p = 6\ kPa)$). The 'Supplementary Data A' file (Directory: Figures/Adsorption) includes all figures for the 25 catalyst configurations with an SS cationic surface. Images for other configurations may be obtained from the authors upon reasonable request.

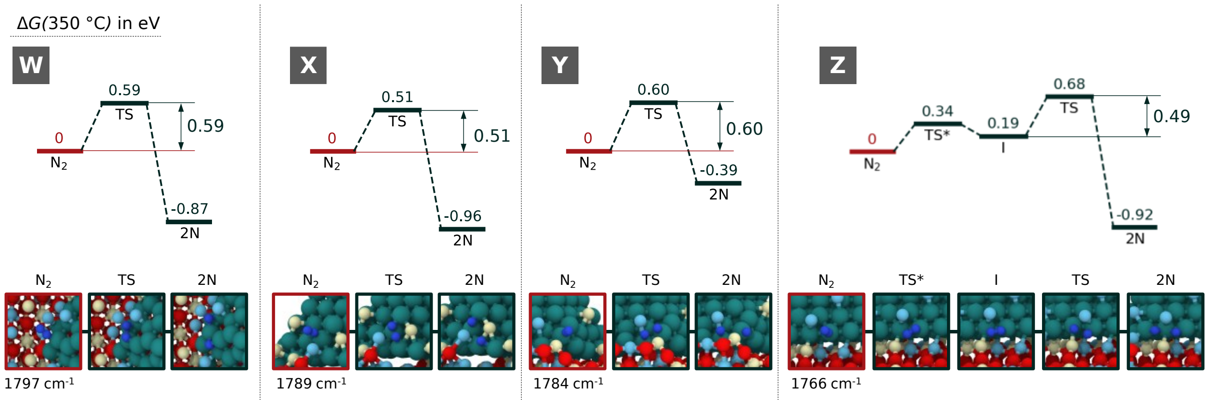

Fig. S5 Additional N_2 dissociation paths with low activation barrier.

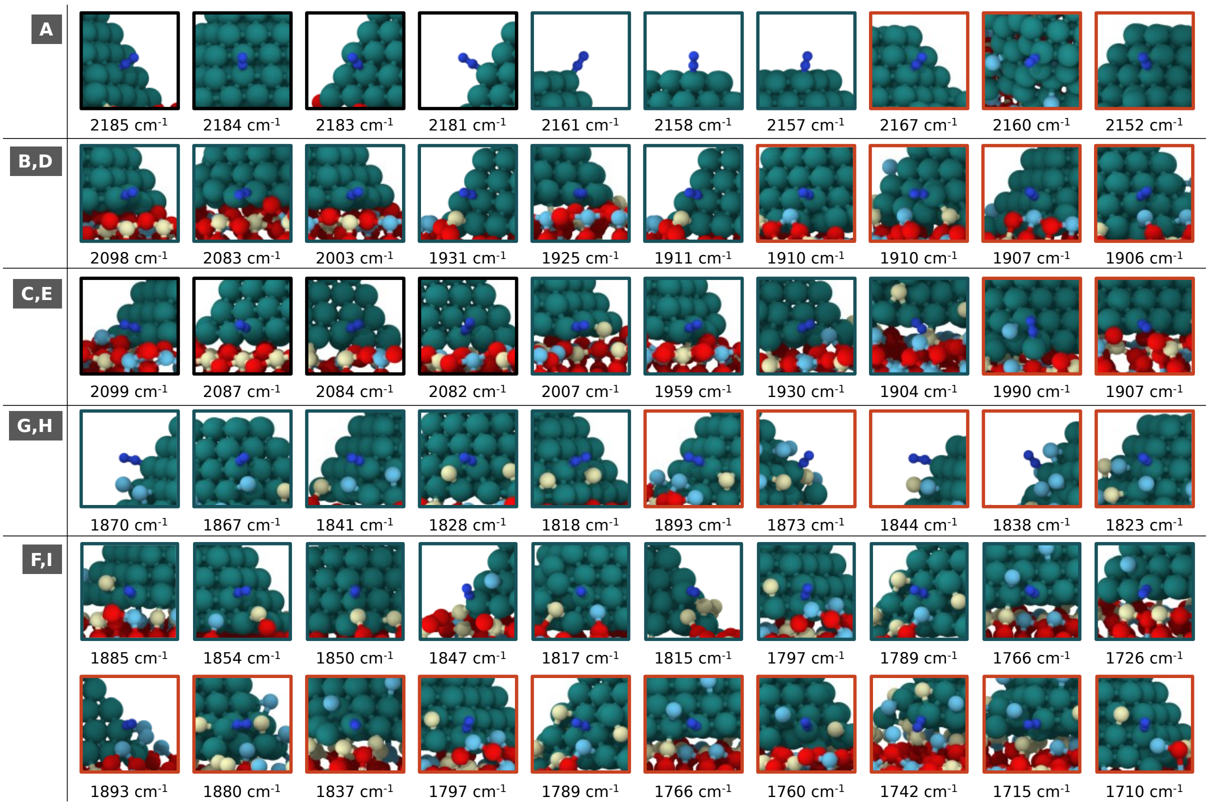

Fig. S6 Examples of identified adsorption states categorised as discussed in Data S5. All adsorption states exhibit high coverage of ≈1.0 at 350 °C and 1 MPa. Frames marked in blue and orange were identified within the configurations assigned to T_{Red} = 500°C and T_{Red} = 650°C, respectively (see Fig. 3 in the article). The 'AdsorptionSites.xlsx' file in the 'Doc' directory of the 'Supplementary Data A' file contains the properties of these and other adsorption states.

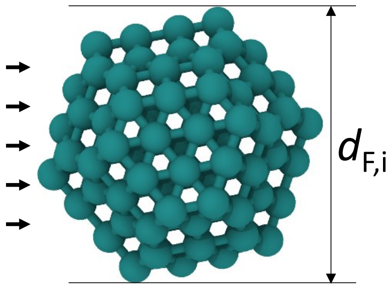

Fig. S7 Illustration of the Feret diameter $d_{F,i}$ for a specified measurement direction i, indicated by the arrows. The measurement direction i is typically defined as any direction perpendicular to the projection direction.

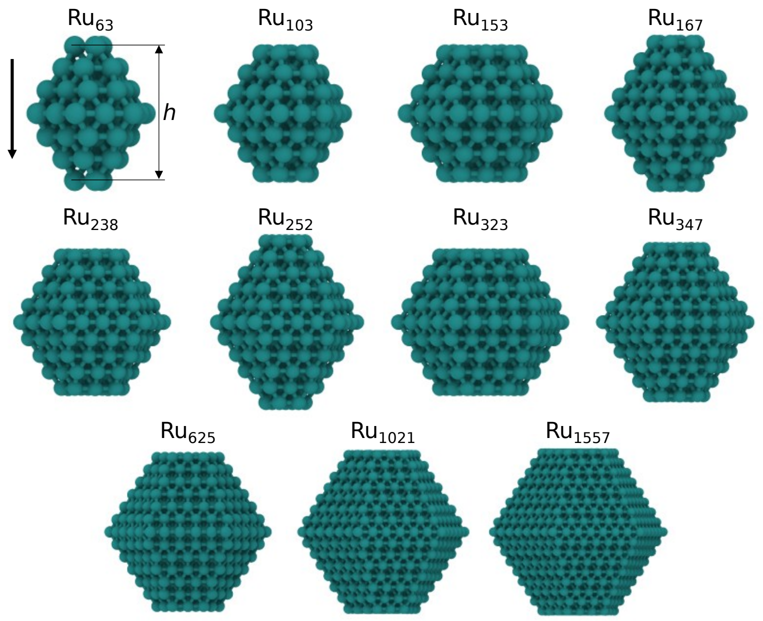

Fig. S8 Various hcp-nanoparticle configurations of different sizes. The arrow indicates the direction perpendicular to the projection area, and *h* represents the height of the nanoparticle. The configurations were taken from the studies of Nanba et al.[19] and Rocabado et al.[21]. The Ru103 configuration was added to provide a more complete size range.

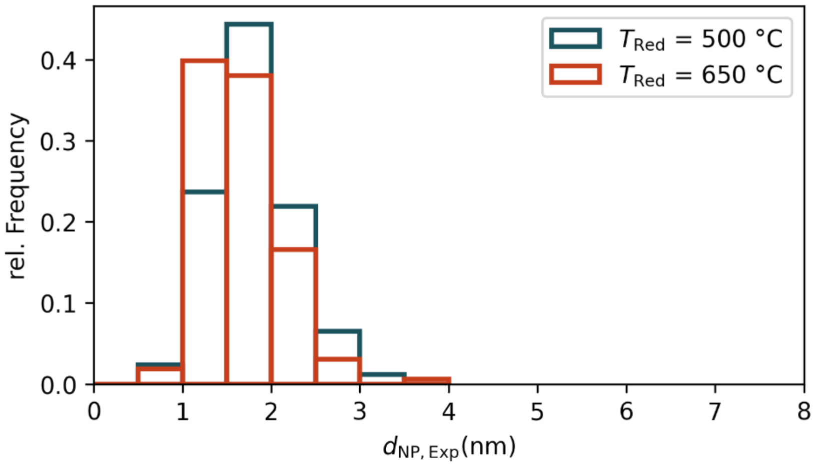

Fig. S9 Experimental ruthenium particle size distributions from STEM images for catalysts reduced at 500 °C (blue) and 650 °C (orange). Values taken from Ogura et al.[2].

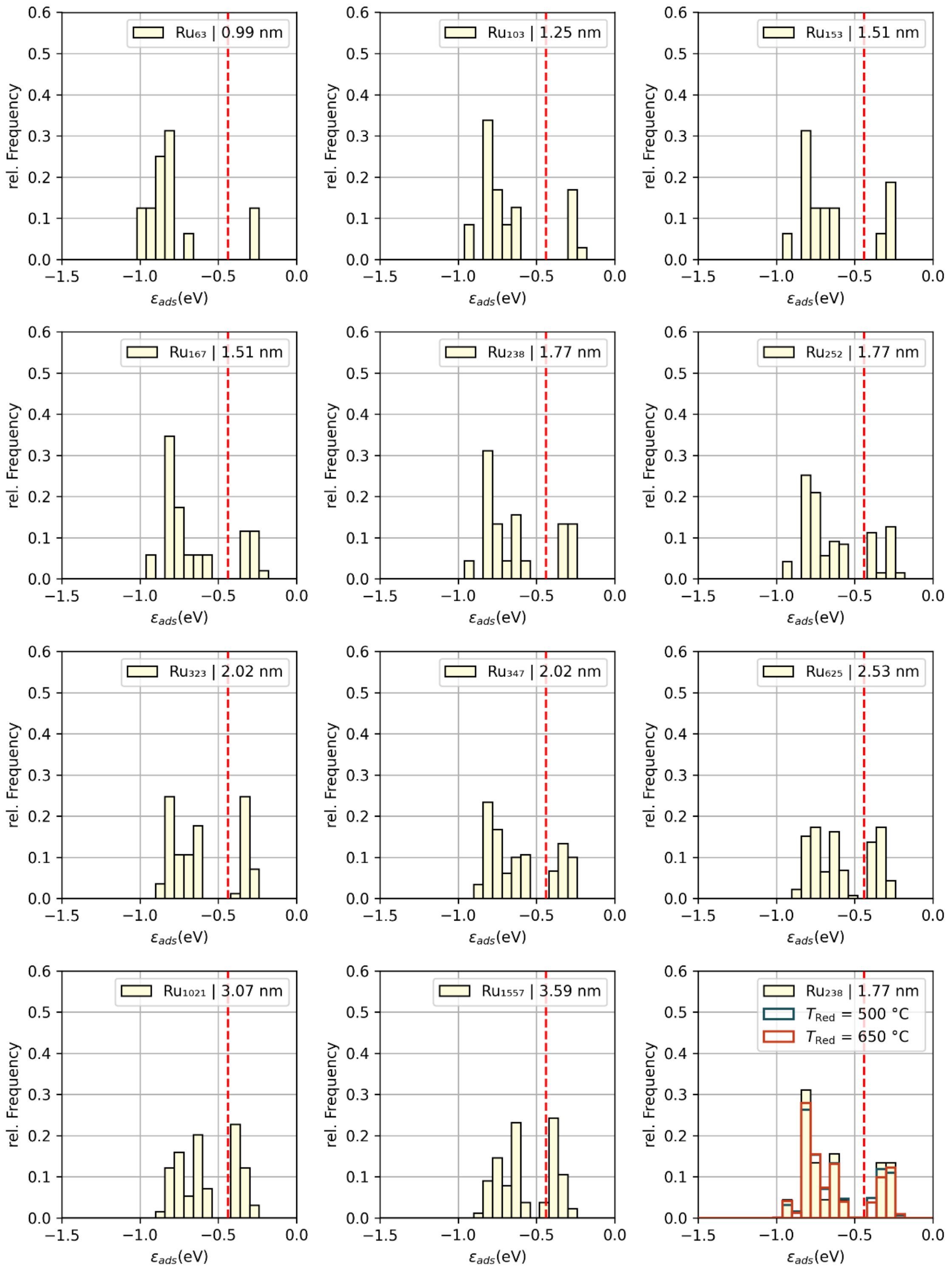

Fig. S10 Calculated histograms of the adsorption energy (ε_{ads}) of N_2 on all on-top sites for various ruthenium hcp-nanoparticle configurations. The final diagram compares the histogram for the Ru_{238} nanoparticle model, used as the basis for this study, with the average histograms constructed by considering the experimental size distribution reported by Ogura et al.[2] for Ru/$La_{0.5}Ce_{0.5}O_{1.75-x}$ catalysts reduced at 500 °C (blue) and 650 °C (orange). The vertical red line represents the calculated value for the N_2 on-top adsorption on the ruthenium slab as a reference.

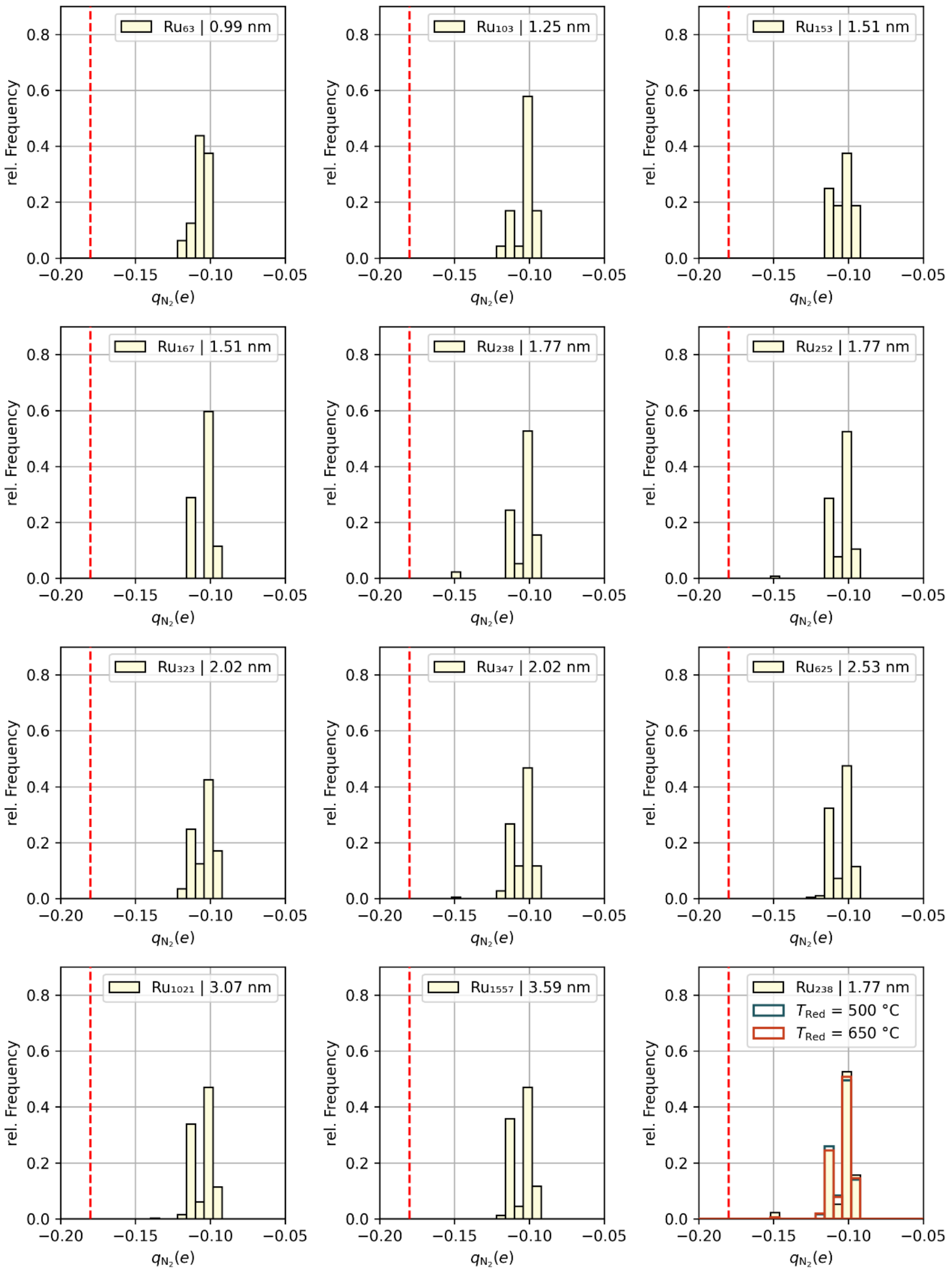

Fig. S11 Calculated histograms of the charge (q_{N_2}) of N_2 molecules adsorbed on all on-top sites for various ruthenium hcp-nanoparticle configurations. The final diagram compares the histogram for the Ru_{238} nanoparticle model, used as the basis for this study, with the average histograms constructed by considering the experimental size distribution reported by Ogura et al.[2] for Ru/$La_{0.5}Ce_{0.5}O_{1.75-x}$ catalysts reduced at 500 °C (blue) and 650 °C (orange). The vertical red line represents the calculated value for the N_2 on-top adsorption on the ruthenium slab as a reference.

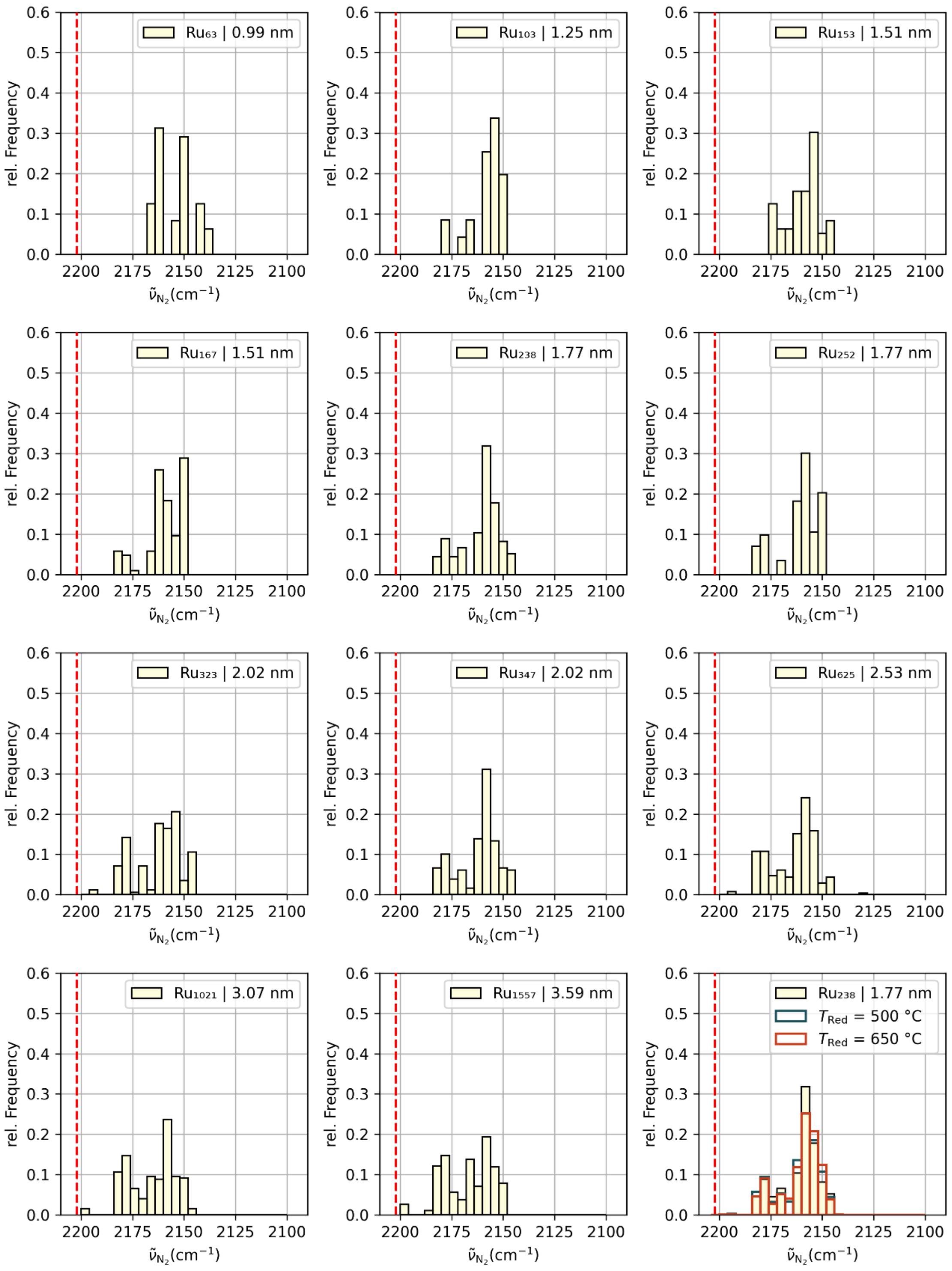

Fig. S12 Calculated histograms of the wavenumber ($\tilde{\nu}_{N_2}$) of the stretching mode of N_2 molecules adsorbed on all on-top sites for various ruthenium hcp-nanoparticle configurations. The final diagram compares the histogram for the Ru_{238} nanoparticle model, used as the basis for this study, with the average histograms constructed by considering the experimental size distribution reported by Ogura et al.[2] for $Ru/La_{0.5}Ce_{0.5}O_{1.75\text{-}x}$ catalysts reduced at 500 °C (blue) and 650 °C (orange). The vertical red line represents the calculated value for the N_2 on-top adsorption on the ruthenium slab as a reference.

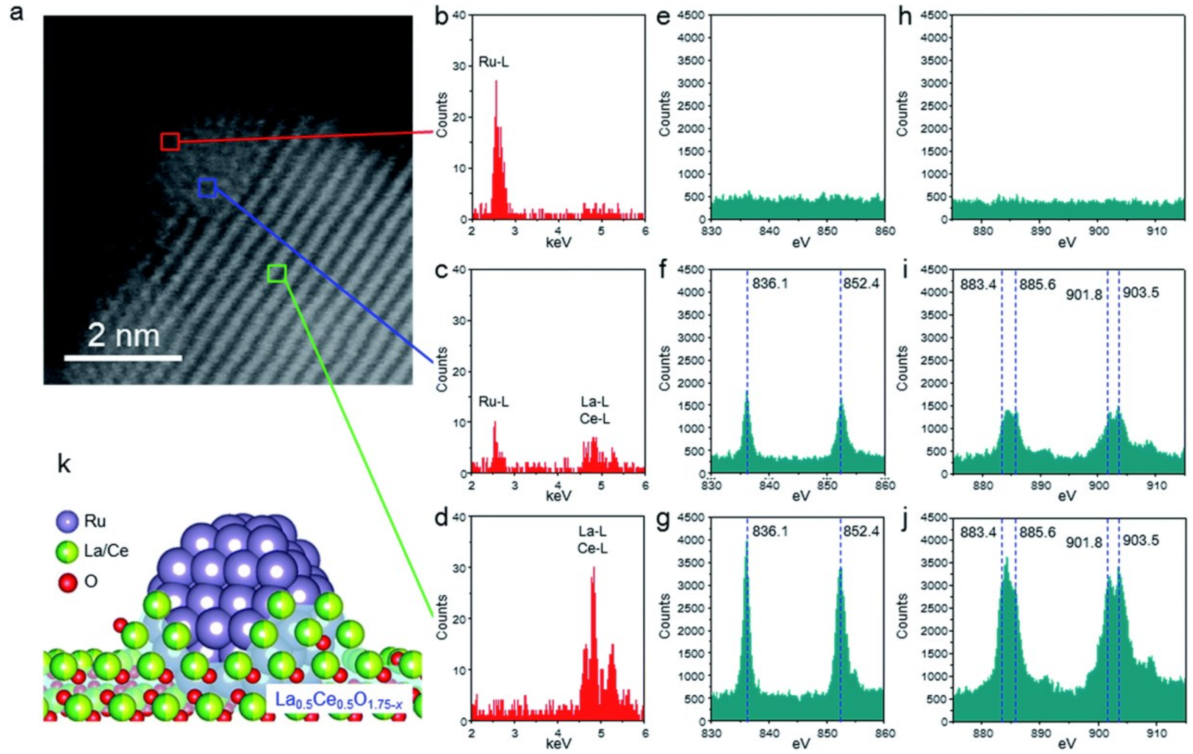

Fig. S13 High-magnification (a) HAADF-STEM image, (b)–(d) EDX spectra, and (e)–(j) EEL spectra of the $Ru/La_{0.5}Ce_{0.5}O_{1.75-x}$ catalyst reduced at 650°C without air exposure. The EDX spectra correspond to the regions marked by red, blue, and green squares in the HAADF-STEM image. EEL spectra of the La $M_{4,5}$ (e)–(g) and Ce $M_{4,5}$ (h)–(j) edges are shown for the areas highlighted by red, blue, and green squares. (k) Illustration of the $Ru/La_{0.5}Ce_{0.5}O_{1.75-x}$ structure. Reproduced from Y. Ogura, K. Sato, S. Miyahara, Y. Kawano, T. Toriyama, T. Yamamoto, S. Matsumura, S. Hosokawa and K. Nagaoka, Chem. Sci., 2018, 9, 2230 DOI: 10.1039/C7SC05343F with permission from the Royal Society of Chemistry (CC by 3.0).

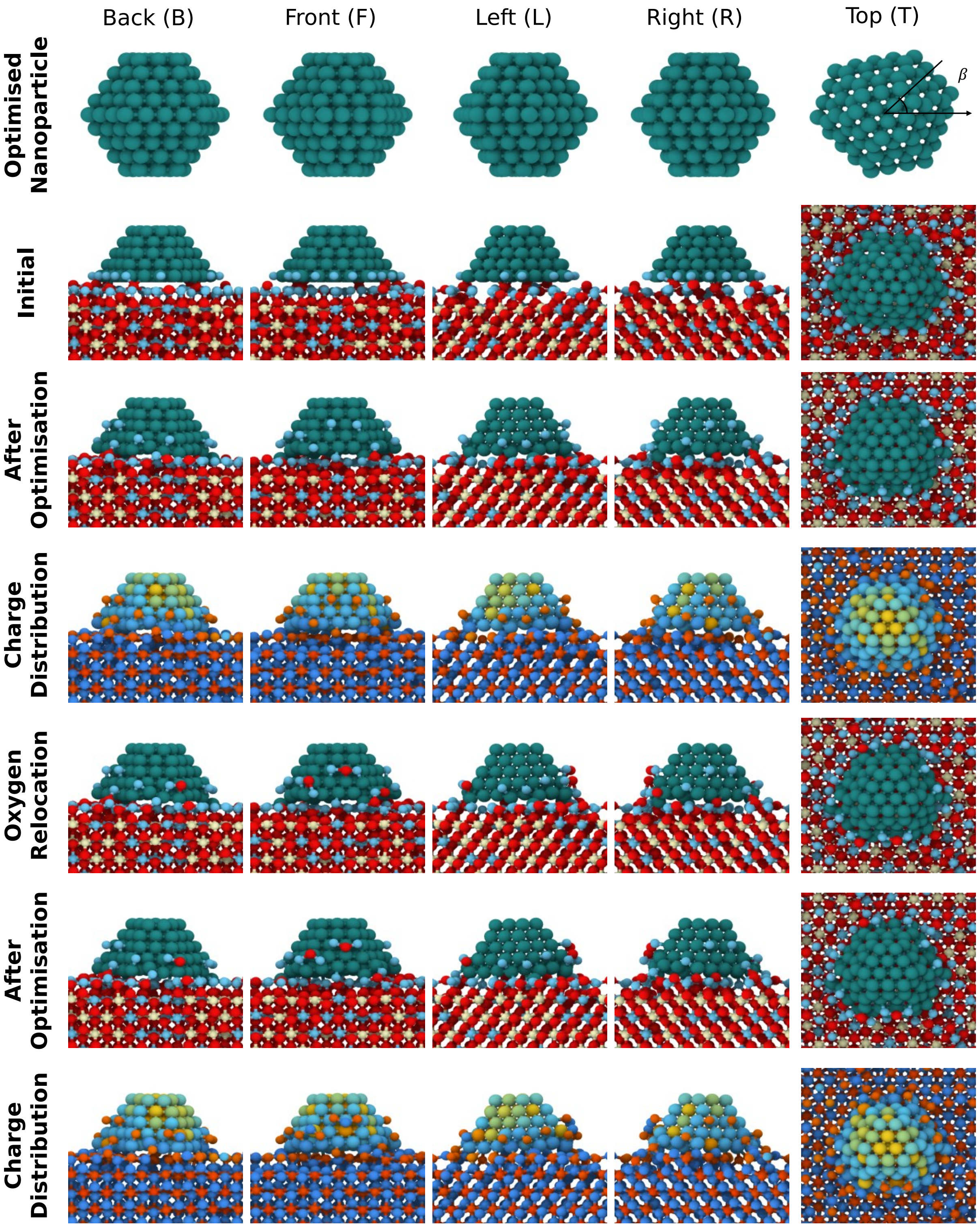

Fig. S14 Optimised Ru_{238} HCP nanoparticle structure including the definition of the angle β and frames for the configurations labelled 165-La-1-1-xO25-1Cat75 and 165-La-1-1-xO25-1CatO75 (see Data S7 for details).

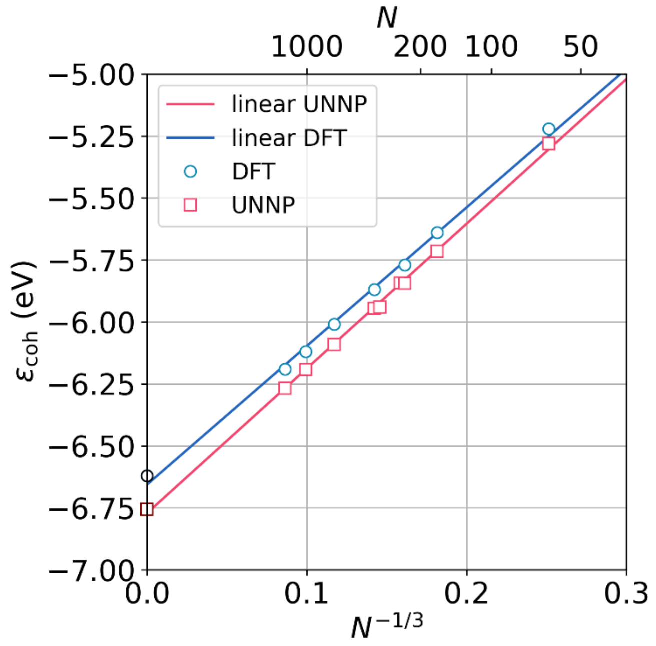

Fig. S15 Calculated cohesive energies ε_{coh} for Ru hcp nanoparticles with various sizes. The values are plotted as a function of the nanoparticle size $N^{-1/3}$. DFT values are from the study of Nanba et al.[19], and UNNP values are from the study of Valadez et al.[18]. Darker symbols represent the values for the bulk phase.

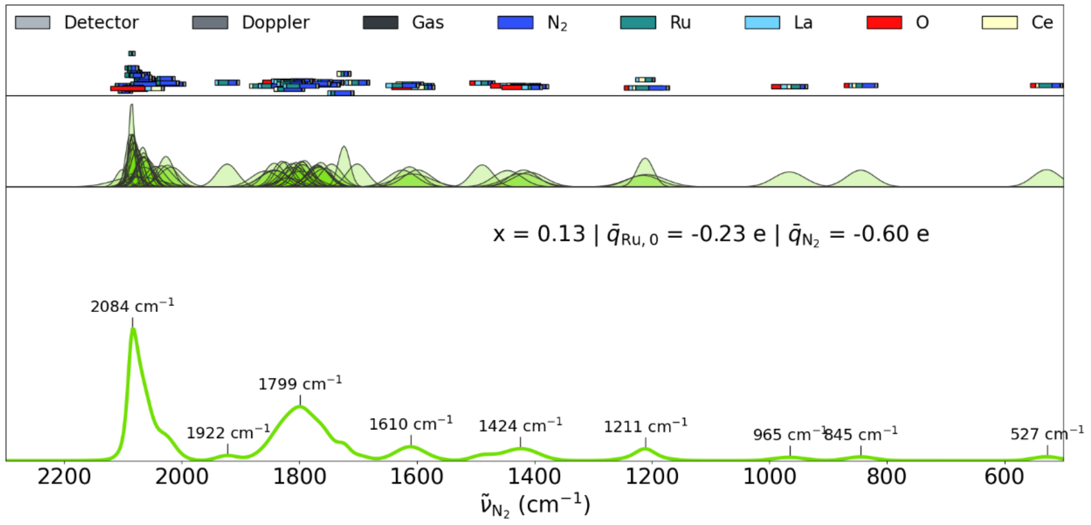

Fig. S16 Wavenumber distribution corresponding to Fig. 3a in the article, but for a ^{15}N isotope.

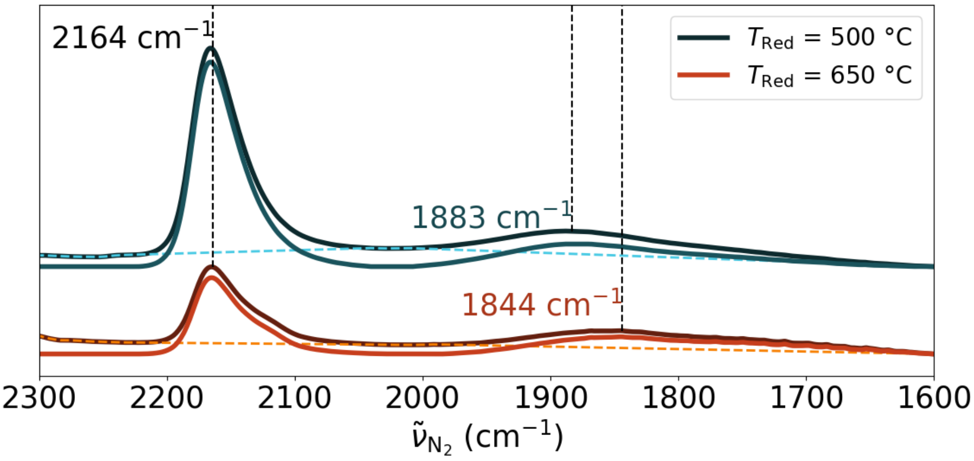

Fig. S17 Measured IR spectra for N_2 adsorption on Ru/$La_{0.5}Ce_{0.5}O_{1.75-x}$ catalysts reduced at 500 °C and 650 °C[2] together with the baseline and resulting baseline-corrected IR spectra (light colours).

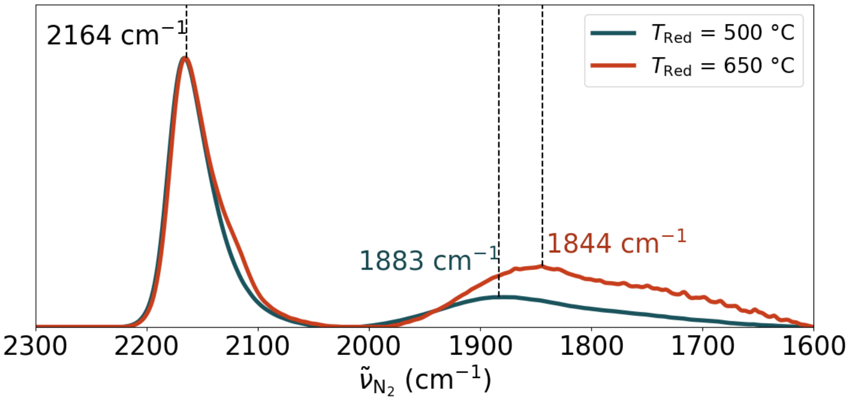

Fig. S18 Baseline-corrected and independently normalised IR spectra for N_2 adsorption on Ru/$La_{0.5}Ce_{0.5}O_{1.75-x}$ catalysts reduced at 500 °C and 650 °C[2].

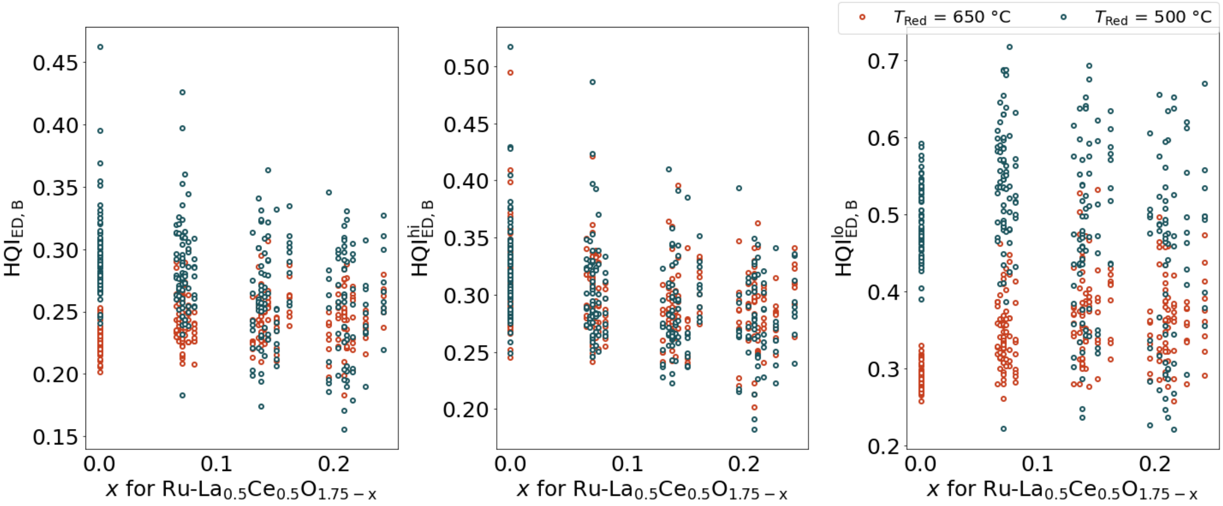

Fig. S19 $HQI_{ED,B}$ between all simulated wavenumber distributions and the post-processed experimental wavenumber spectra depicted in Fig. S17, where B denotes the post-processing method (hi: $\tilde{\nu}_{N_2} \geq 2050\ \mathrm{cm}^{-1}$; lo: $\tilde{\nu}_{N_2} < 2050\ \mathrm{cm}^{-1}$).

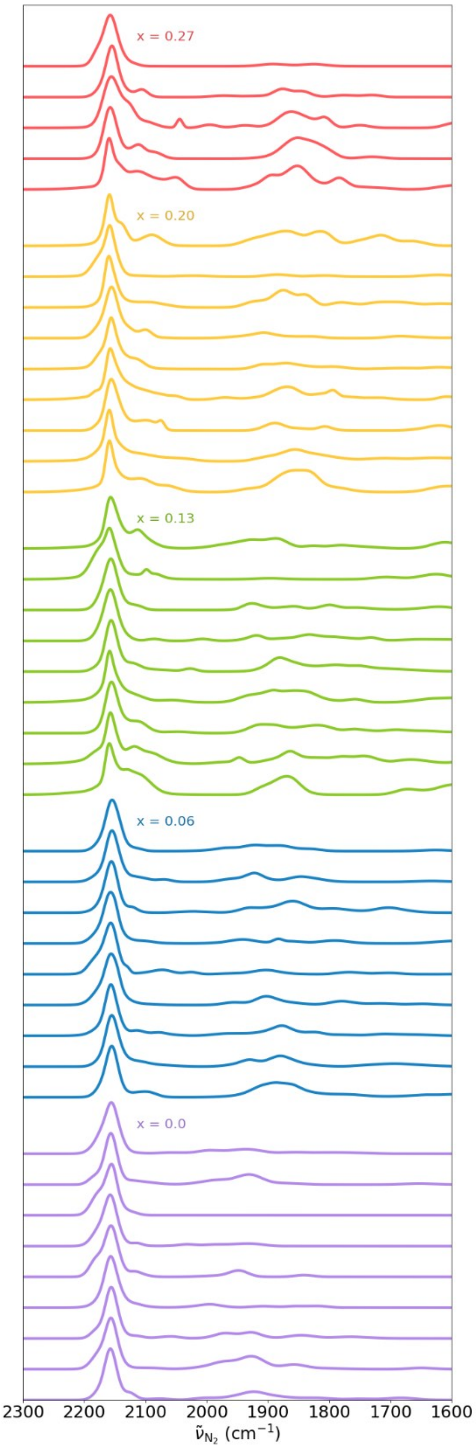

Fig. S20 Calculated normalised wavenumber distributions for all catalyst configurations corresponding to one of the five cationic surfaces with solid–solution composition depending on the reduction degree x. The wavenumber distributions indicated in each colour are arranged in descending order based on the 'artificial SMSI' condition, from low to high values.

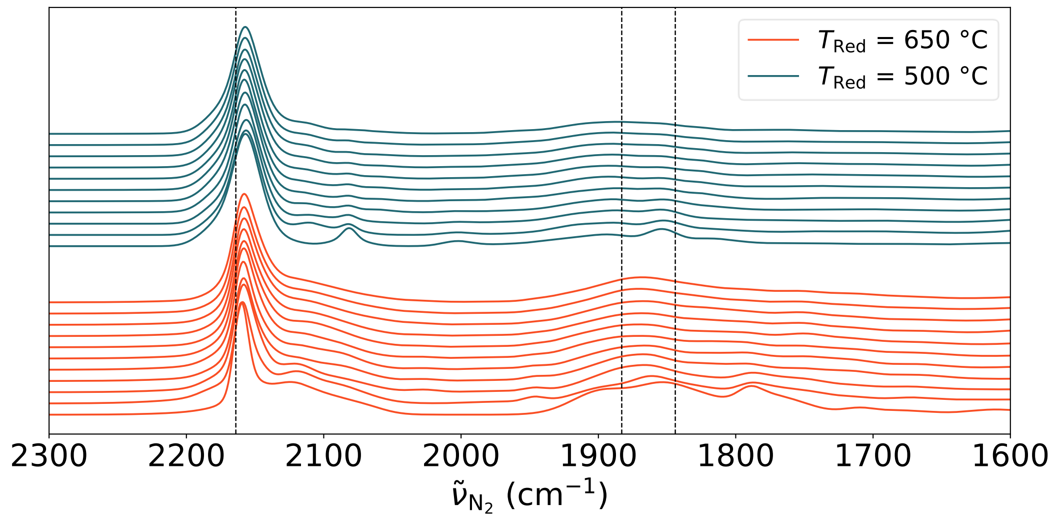

Fig. S21 Cumulative averages of the wavenumber distribution obtained by averaging the configurations in descending order of their $\mathrm{HQI}_{\mathrm{B}}^{\mathrm{lo}}$ value compared to the experimental spectra. The lowest spectrum for each case corresponds to the cumulative average by considering only one distribution, the next spectrum to the cumulative average of two distributions, and so on.

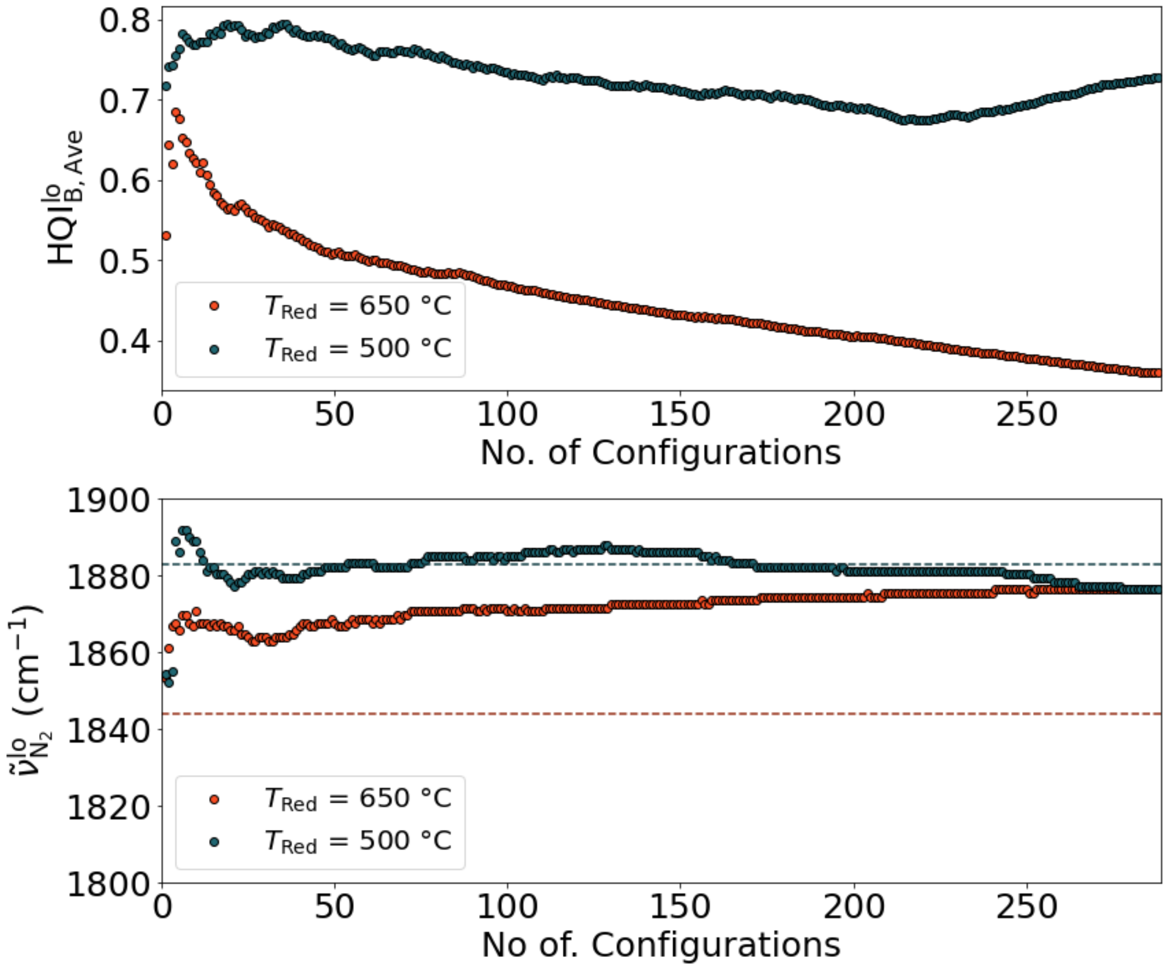

Fig. S22 Upper diagram: Calculated $\mathrm{HQI}_{\mathrm{B,Ave}}^{\mathrm{lo}}$ values for the cumulative averages of the wavenumber distribution averaged in descending order of the $\mathrm{HQI}_{\mathrm{B,Ave}}^{\mathrm{lo}}$ (TRed = 500 °C) values and of the $\mathrm{HQI}_{\mathrm{B,Ave}}^{\mathrm{lo}}$(TRed = 650 °C) values. Lower diagram: resulting low-wavenumber peak for the cumulative averages. The dashed lines denote experimental values[2].

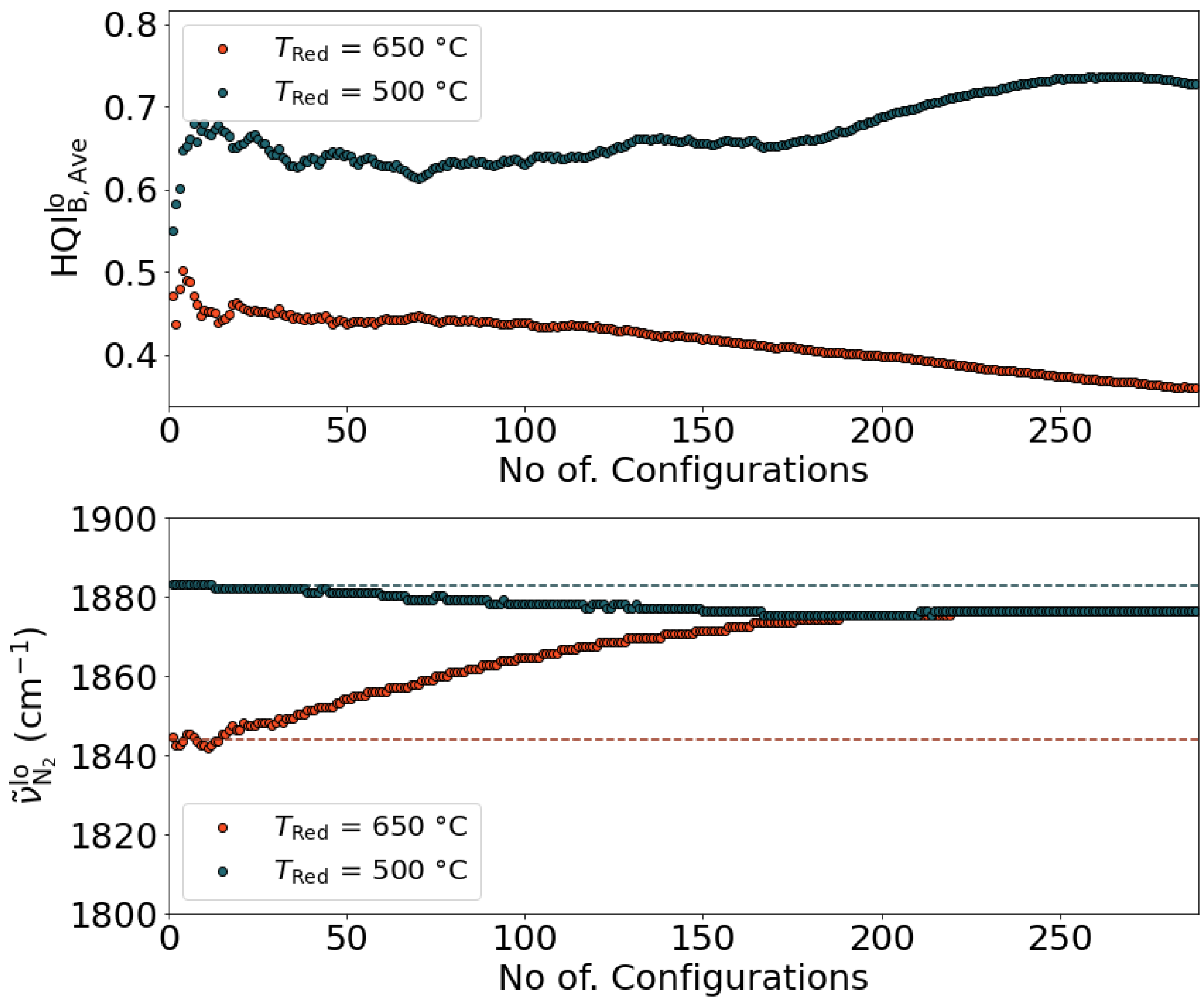

Fig. S23 Upper diagram: Calculated $\mathrm{HQI}^{\mathrm{lo}}_{\mathrm{B,Ave}}$ values for the cumulative averages of the wavenumber distribution averaged in ascending order of $\Delta\tilde{\nu}^{\mathrm{lo}}_{\mathrm{N_2}}$($T_{\mathrm{Red}}$ = 500 °C) values and $\Delta\tilde{\nu}^{\mathrm{lo}}_{\mathrm{N_2}}$($T_{\mathrm{Red}}$ = 650 °C) values. Lower diagram: resulting peak of the low wavenumber for the cumulative averages. The dashed lines denote the experimental values[2].

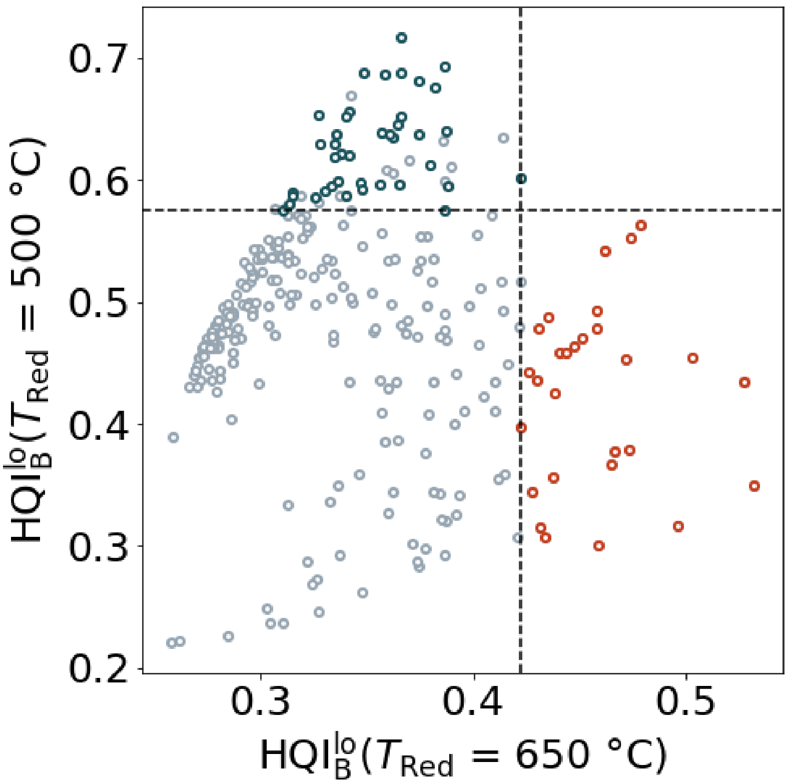

Fig. S24 Calculated $\mathrm{HQI}_\mathrm{B}^\mathrm{lo}$ value for all configurations. The markers for the configurations used to calculate the average for T_Red = 500 °C are coloured blue, where the horizontal dashed line denotes the lowest limit for the $\mathrm{HQI}_\mathrm{B}^\mathrm{lo}$. The marker for the pre-selected configurations used for optimisation with the GA for T_Red = 650 °C are coloured orange, where the vertical dashed line denotes the chosen selection limit.

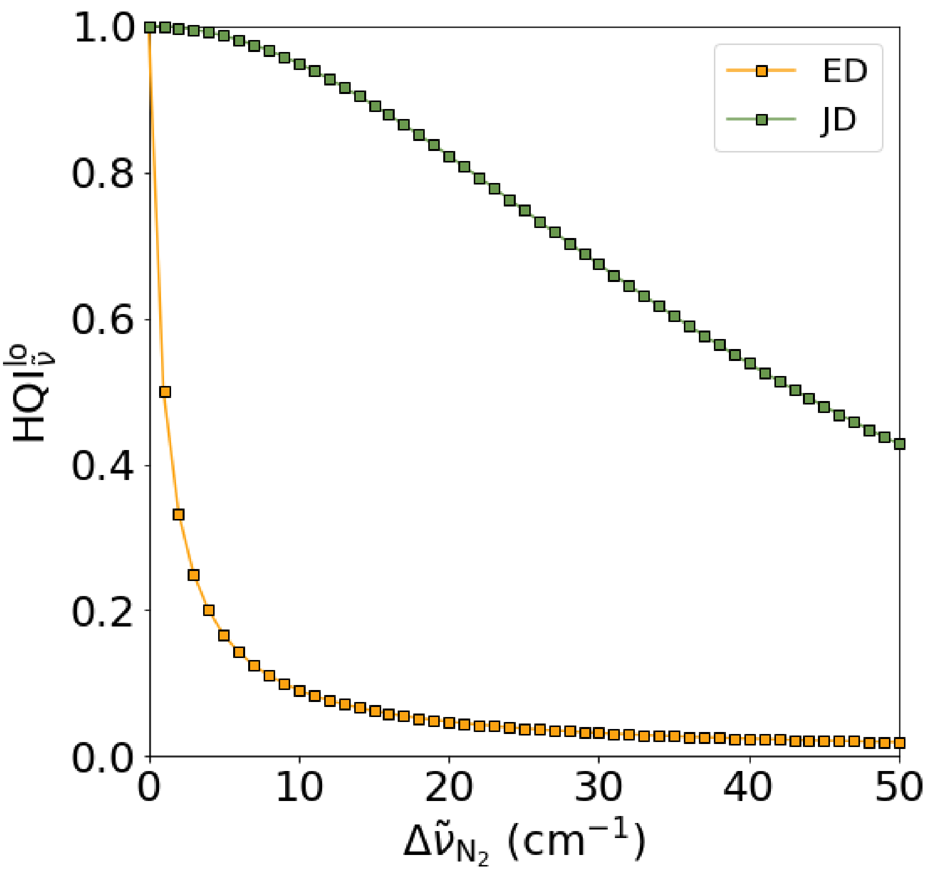

Fig. S25 $HQI_{\tilde{\nu}}^{lo}$ as a function of $\Delta\tilde{\nu}_{N_2}^{lo}$.

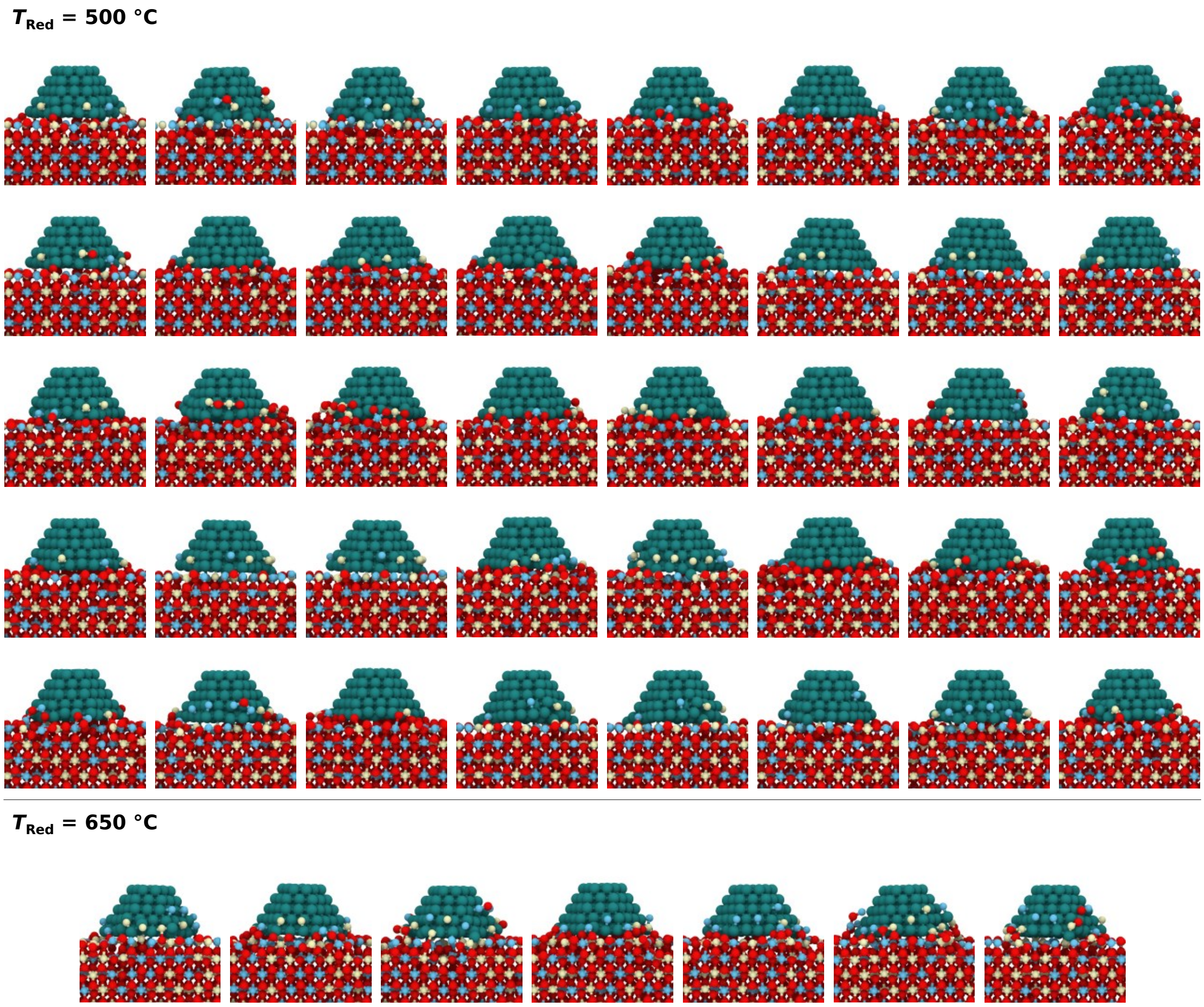

Fig. S26 Set of catalyst configurations for which the calculated wavenumber distributions for the N_2 stretching mode, on average, reproduce the experimental N_2 IR spectra for catalyst reduction temperatures of T_{Red} = 500 °C (13 configurations) and T_{Red} = 650 °C (8 configurations).

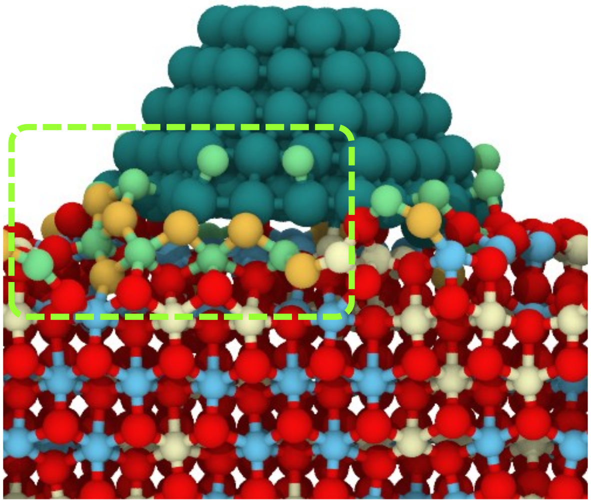

Fig. S27 Displaced Ce and La atoms (in green) and O atoms (in yellow) identified by Wigner-Seitz defect analysis for a configuration included in the configuration set corresponding to T_{Red} = 650 °C. The marked region is an example containing various kinds of displaced atoms: atoms displaced to the second atomic layer of the ruthenium nanoparticle; atoms displaced at the cationic surface near the nanoparticle due to interactions between the nanoparticle and the support; and atoms displaced due to surface reconstruction (uppermost left part of the marked region).

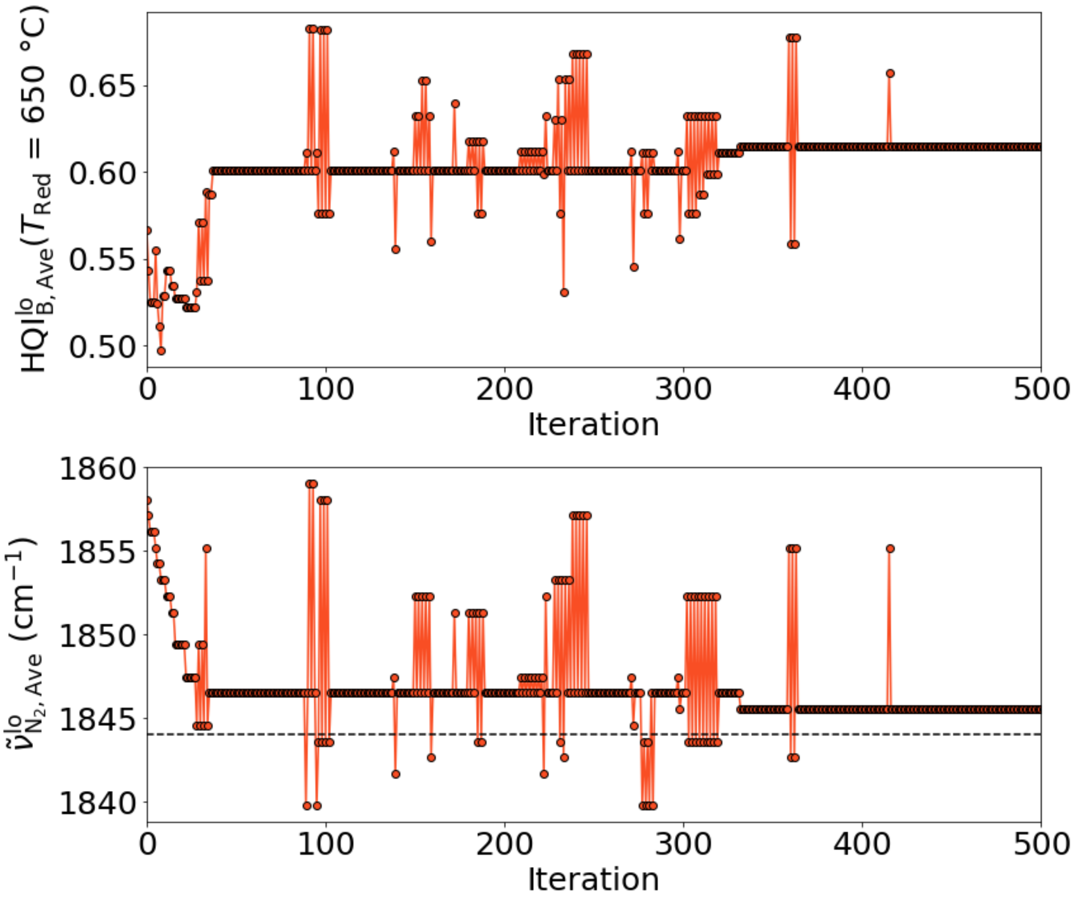

Fig. S28 Calculated values for $\mathrm{HQI}^{\mathrm{lo}}_{\mathrm{B,Ave}}$ for T_{Red} = 650 °C (upper figure) and $\tilde{\boldsymbol{\nu}}^{\mathbf{lo}}_{\mathbf{N_2}}$ during optimisation using the GA for the first 100 iterations.

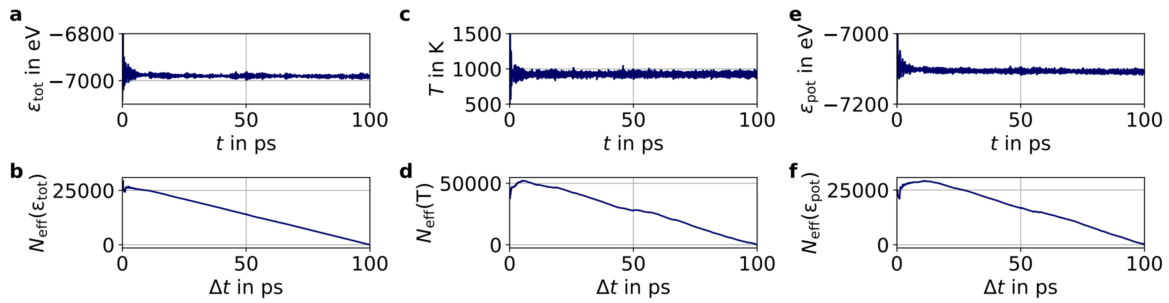

Fig. S29 Changes in (a) total energy ε_{tot}, (c) temperature T, and (e) potential energy ε_{pot} over the simulation time t. b, d, f, Corresponding number of uncorrelated samples N_{eff} over the equilibration timespan Δt. All depictions are given as an example for a slab with a Ce surface and represent all other configurations. The system is considered to reach equilibrium over 100 ps because the number of uncorrelated samples is highest within this timespan and linearly approaches zero afterwards[25].

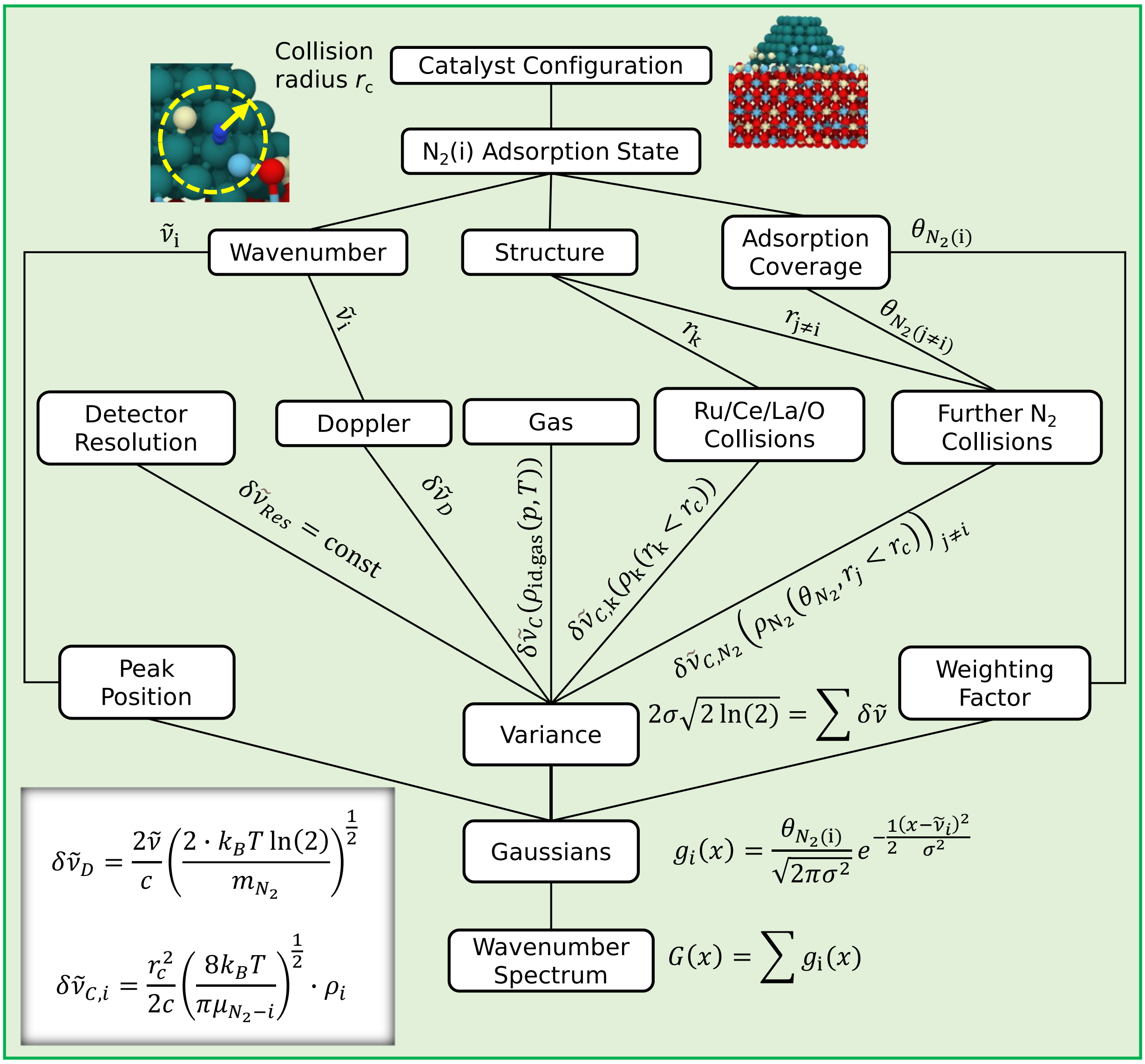

Fig. S30 Methods used to calculate the wavenumber distribution of adsorbed N_2 molecules on each catalyst configuration.

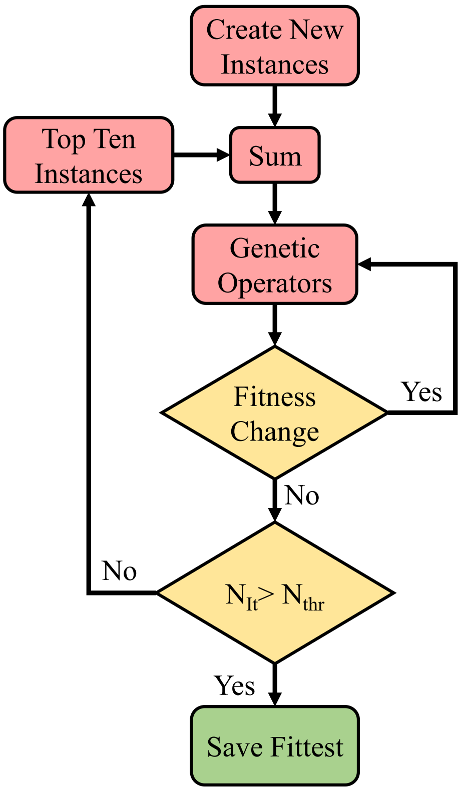

Fig. S31 Flow chart of the GA used in this study.

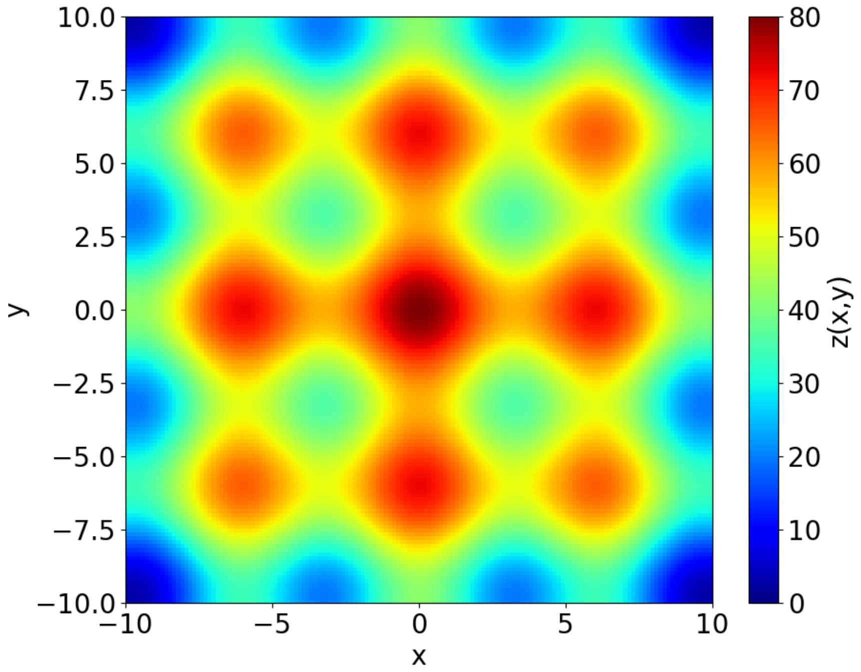

Fig. S32 Colour map for the calculated values of the function z(x,y) used for testing the GA.

Supplemental Tables

Table S1. Comparison of the calculated Gibbs energy barriers for the dissociation of a N_2 molecule on a Ru slab (on-top) toward two hollow sites (2N) using the UNNP and DFT values. The Gibbs energy is defined as $\Delta G = G(\text{TS})\text{-}G(\text{N}_2)$, similar to the definition in Fig. 3d in the article. The DFT values correspond to $\Delta G = \Delta G^{\ddagger}+\Delta G_r$ because the values are given for $\Delta G^{\ddagger} = G(\text{TS})\text{-}G(2\text{N})$ and $\Delta G_r = G(2\text{N})\text{-}G(\text{N}_2)$ in the literature[26]. The UNNP calculations were performed for a 2 × 2 × 6 Ru slab (HCP) with a 0001 surface by fixing the four lowest slab layers. The DFT calculations were performed for a 2 × 2 × 2 Ru slab (HCP) by fixing the lowest layer[26].

T in K	ΔG_{UNNP} (eV)	ΔG_{DFT} (eV)
100	1.56	1.66
300	1.60	1.70
700	1.73	1.80
1,000	1.83	1.88
1,400	1.97	1.99

Table S2 Calculated values for the diameter of the surface-equivalent sphere (d_S), the diameter of the projection area-equivalent circle (d_P), the average and maximum Feret diameters (d_F and $d_{F,max}$, respectively) and the height (h) for various nanoparticle configurations.

N_{Ru}	d_S in nm	d_P in nm	d_F in nm	$d_{F,max}$ in nm	h in nm
63	0.959	0.950	0.995(13)	1.037	1.236
103	1.175	1.191	1.253(16)	1.305	1.358
153	1.379	1.444	1.511(19)	1.575	1.268
167	1.438	1.445	1.511(19)	1.575	1.676
238	1.646	1.687	1.771(22)	1.846	1.688
252	1.696	1.687	1.771(22)	1.846	2.098
323	1.852	1.939	2.029(26)	2.115	1.691
347	1.911	1.940	2.030(26)	2.116	2.111
625	2.386	2.436	2.539(33)	2.657	2.537
1021	2.857	2.933	3.070(40)	3.201	2.964
1557	3.330	3.429	3.592(46)	3.745	3.389

Table S3 Calculated values for various HQIs between the experimental spectra, baseline corrected, and normalised. Case A in the table corresponds to the spectra in Fig. S17 and case B to the spectra in Fig. S18. The values are calculated for each case by accounting on the whole spectrum, only the high-wavenumber region (hi), or only the low-wavenumber region (lo).

Case	HQI_{PCC}	HQI_{ED}	HQI_{RMSD}	HQI_{JD}
A	0.97	0.23	0.89	0.03
A (hi: $\tilde{\nu}_{N_2} \geq 2050\ \text{cm}^{-1}$)	0.99	0.23	0.83	0.03
A (lo: $\tilde{\nu}_{N_2} < 2050\ \text{cm}^{-1}$)	0.85	0.70	0.98	0.17
B	0.97	0.37	0.94	0.03
B (hi: $\tilde{\nu}_{N_2} \geq 2050\ \text{cm}^{-1}$)	0.99	0.63	0.96	0.40
B (lo: $\tilde{\nu}_{N_2} < 2050\ \text{cm}^{-1}$)	0.85	0.39	0.93	0.03

Table S4 Peaks at low and high wavenumbers in the experimental IR spectra depicted in Fig. 3b in the article.

T_{Red} in °C	$\tilde{\nu}^{hi}_{N_2,Exp}$ (cm^{-1})	$\tilde{\nu}^{lo}_{N_2,Exp}$ (cm^{-1})
500	2,164	1,883
650	2,164	1,846

Table S5 Average properties of the catalyst configuration sets for which the calculated wavenumber distributions for the N_2 stretching mode, on average, reproduce the experimental N_2 IR spectra[2] for catalyst reduction temperatures of T_{Red} = 500 °C and T_{Red} = 650 °C. x denotes the reduction degree, $q_{Ru,0}$ the average charge of the Ru atoms on the nanoparticle surface layer. $\boldsymbol{N_{V_O^{\bullet\bullet}(S)}}$ and $N_{Ce(S)+La(S)}$ represent the number of oxygen vacancies and Ce and La cations on the cationic surface (S), respectively. $N_{i=Ce(D),La(D),O(D)}$ denotes the number of displaced Ce, La, O atoms from their ideal lattice positions.

T_{Red}	x	$q_{Ru,0}$ in e	$N_{V_O^{\bullet\bullet}(S)}$	$N_{Ce(S)+La(S)}$	$N_{La(D)}$	$N_{Ce(D)}$	$N_{O(D)}$
500 °C	0.119(20)	−0.137(23)	35(6)	57(1)	4(1)	4(2)	4(4)
650°°C	0.160(46)	−0.225(46)	32(16)	49(2)	8(2)	7(2)	4(3)

Table S6 Average bond distance r_{i-j} and Warren-Cowley Parameter α_{ij} between the cations (i,j = Ce,La) for the catalyst configuration sets for which the calculated wavenumber distributions for the N_2 stretching mode, on average, reproduce the experimental N_2 IR spectra[2] for catalyst reduction temperatures of T_{Red} = 500 °C and T_{Red} = 650 °C.

	T_{Red}	Ce-Ce	Ce-La	La-Ce	La-La
r_{i-j} in Å	500 °C	3.9831(21)	3.9978(13)		3.9931(20)
	650°°C	3.9803(50)	3.9977(51)		3.9953(39)
α_{ij}	500 °C	-0.0001(24)	-0.0039(21)	-0.0042(36)	-0.0005(37)
	650°°C	0.0016(36)	-0.0086(58)	-0.005(13)	0.001(13)

Table S7 Number of Ce/La/O ions and oxygen vacancies (V_O) considered in the local environment of the adsorbed nitrogen molecule depicted in Fig. 1 in the article, depending on the cut-off radius ($N_i(r_{cut})$ where i = N_{Ce+La}|$N_{Ce(D)+La(D)}$|N_O|N_{VO} and D represents ions displaced from its original position within the metal oxide) The wavenumber $\boldsymbol{\tilde{\nu}_{N_2}}$ and position of the N_2 molecule in z direction relative to configuration c (which shows the lowest position) are also provided.

States	A	B	C	D	E	F	G	H	I
$\tilde{\nu}_{N_2}$ (cm^{-1})	2161	2090	2066	1967	1917	1883	1846	1844	1751
z-z_c (Å)	8.17	4.14	0.0	2.05	0.0	2.35	4.34	1.59	2.42
N_i(2 Å)					0|0|0|0	0|0|0|0	0|0|0|0	0|0|0|0	0|0|0|0
N_i(3 Å)			0|0|0|0	0|0|0|0	2|0|0|2	2|2|0|0	2|2|0|0	2|2|0|0	2|2|0|0
N_i(4 Å)		0|0|0|0	2|0|4|2	2|2|0|0	2|0|6|2	2|2|0|0	2|2|0|0	2|2|0|0	2|2|2|0
N_i(5 Å)		6|4|2|0	6|0|6|4	2|2|6|0	10|0|12|6	4|4|2|0	4|4|0|0	4|4|0|2	6|4|2|0
N_i(6 Å)	0|0|0|0	6|4|2|0	12|0|20|8	4|2|10|0	14|0|22|10	6|4|4|2	6|6|0|0	8|8|0|8	8|6|6|2

Table S8 Angular variations of the Ru nanoparticle on the $La_{0.5}Ce_{0.5}O_{1.75-x}$ slab (see Fig. S14 for the definition of angle β) and corresponding interfacial energy ε_{inter} for different catalyst configurations.

	Ce-1	La-1	SL-1	SS-1
β (°)	$\varepsilon_{\text{inter}}$ (eV)	$\varepsilon_{\text{inter}}$ (eV)	$\varepsilon_{\text{inter}}$ (eV)	$\varepsilon_{\text{inter}}$ (eV)
75	-133.33	-132.33	-133.07	-126.42
105	-116.79	-135.11	-137.93	-131.94
135	-133.79	-129.65	-132.12	-127.40
165	-140.58	-137.59	-137.53	-132.51
195	-133.33	-132.33	-133.07	-126.42

Table S9 Cell lengths *a*, *b*, and *c* calculated using DFT, as provided in the Materials Project Database[20], compared with values obtained using the UNNP for various bulk systems containing La, Ce, Ru, and O. The cell lengths may not necessarily match the lattice constants, as we directely used the cell size and shape from the database.

Formula	Crystal System	*a* (Å)		*b* (Å)		*c* (Å)	
		DFT	UNNP	DFT	UNNP	DFT	UNNP
La_7Ru_3	Orthorhombic	6.76	6.82	7.41	7.35	23.78	24.13
$LaRu_2$	Cubic	7.68	7.74	7.68	7.75	7.68	7.74
La_3Ru	Cubic	6.60	6.73	7.39	7.29	10.14	10.21
La_5Ru_2	Monoclinic	16.91	17.25	6.84	6.80	7.30	7.32
LaO_2	Monoclinic	4.31	4.01	3.93	4.01	6.36	6.07
LaO_3	Hexagonal	5.43	6.83	5.43	4.02	4.53	4.17
La_2O_3	Cubic	11.36	11.37	11.36	11.37	11.36	11.37
La_2O_3	Trigonal	3.93	3.92	3.93	3.92	6.11	6.15
$La_2Ce_2O_7$	Cubic	11.18	11.29	11.18	11.29	11.18	11.29
$La_3Ru_3O_{11}$	Cubic	9.54	9.54	9.54	9.54	9.54	9.54
$La_7Ru_3O_{18}$	Trigonal	9.91	9.93	9.91	9.93	56.74	56.80
La_3RuO_7	Orthorhombic	7.41	7.80	11.21	11.12	7.62	7.61
La_3RuO_7	Monoclinic	8.84	9.01	5.70	5.81	12.54	12.55
$La_4Ru_6O_{19}$	Cubic	9.09	9.10	9.09	9.10	9.09	9.10
La_2RuO_5	Triclinic	5.88	5.97	8.01	7.79	9.25	9.51
La_2RuO_5	Monoclinic	7.93	7.79	5.91	5.97	9.31	9.51
$La_8Ru_4O_{21}$	Hexagonal	9.95	9.94	9.95	9.94	10.92	11.01
$LaRuO_3$	Orthorhombic	5.48	5.60	5.91	5.75	7.92	7.96
RuO_2	Tetragonal	4.48	4.52	4.48	4.52	3.11	3.13
RuO_2	Cubic	4.84	4.88	4.84	4.88	4.84	4.88
RuO_4	Monoclinic	9.22	10.03	4.37	4.65	8.36	8.74
RuO_4	Cubic	8.47	8.98	8.47	8.98	8.47	8.98
CeO	Cubic	4.97	5.00	4.97	5.00	4.97	5.00
Ce_7O_{12}	Trigonal	10.34	10.43	10.34	10.43	9.58	9.69
CeO_2	Cubic	5.47	5.54	5.47	5.54	5.47	5.54
CeO_2	Cubic	5.47	5.54	5.47	5.54	5.47	5.54
Ce_2O_3	Cubic	11.19	11.13	11.19	11.13	11.19	11.13
Ce_2O_3	Trigonal	3.87	3.85	3.87	3.85	5.90	6.15
$Ce_{11}O_{20}$	Triclinic	6.73	6.82	6.75	6.88	10.25	10.39
Ce_3Ru	Orthorhombic	6.10	6.10	6.97	6.96	9.76	9.94
$CeRu_2$	Cubic	7.53	7.46	7.53	7.82	7.53	7.44
$Ce_{16}Ru_9$	Trigonal	13.50	13.58	13.50	13.58	22.56	22.61
Ce_4Ru_3	Monoclinic	8.60	8.39	13.26	13.60	5.92	6.17
Ce_7Ru_3	Hexagonal	9.63	9.60	9.63	9.60	6.17	6.22
$LaCeRu_4$	Cubic	7.60	7.67	7.60	7.65	7.60	7.67

Table S10 Cell angles α, β, and γ calculated using DFT, as provided in the Materials Projsect Database[20], compared with values obtained using the UNNP for various bulk systems containing La, Ce, Ru, and O. The cell angles may not necessarily match those of the primitive unit cell, as we directly used the cell size and shape from the database.

Formula	System	α (°)		β (°)		γ (°)	
		DFT	UNNP	DFT	UNNP	DFT	UNNP
La_7Ru_3	Orthorhombic	90.00	90.00	90.00	90.00	90.00	90.00
$LaRu_2$	Cubic	90.00	89.89	90.00	89.89	90.00	90.11
La_3Ru	Cubic	90.00	90.00	90.00	90.00	90.00	90.00
La_5Ru_2	Monoclinic	90.00	90.00	93.64	93.84	90.00	90.00
LaO_2	Monoclinic	90.00	90.00	99.94	90.69	90.00	90.00
LaO_3	Hexagonal	90.00	90.00	90.00	90.00	120.00	104.02
La_2O_3	Cubic	90.00	90.00	90.00	90.00	90.00	90.00
La_2O_3	Trigonal	90.00	90.00	90.00	90.00	120.00	120.00
$La_2Ce_2O_7$	Cubic	90.00	90.03	90.00	90.00	90.00	90.00
$La_3Ru_3O_{11}$	Cubic	90.00	90.00	90.00	90.00	90.00	90.00
$La_7Ru_3O_{18}$	Trigonal	90.00	90.00	90.00	90.00	120.00	120.00
La_3RuO_7	Orthorhombic	90.00	90.00	90.00	90.00	90.00	90.00
La_3RuO_7	Monoclinic	90.00	90.00	104.60	104.29	90.00	90.00
$La_4Ru_6O_{19}$	Cubic	90.00	90.00	90.00	90.00	90.00	90.00
La_2RuO_5	Triclinic	101.14	99.74	91.80	90.00	89.79	90.00
La_2RuO_5	Monoclinic	90.00	90.00	100.42	99.74	90.00	90.00
$La_8Ru_4O_{21}$	Hexagonal	90.00	90.00	90.00	90.00	120.00	120.00
$LaRuO_3$	Orthorhombic	90.00	90.00	90.00	90.00	90.00	90.00
RuO_2	Tetragonal	90.00	90.00	90.00	90.00	90.00	90.00
RuO_2	Cubic	90.00	90.00	90.00	90.00	90.00	90.00
RuO_4	Monoclinic	90.00	90.06	116.49	117.57	90.00	90.01
RuO_4	Cubic	90.00	90.53	90.00	90.59	90.00	90.62
CeO	Cubic	90.00	90.00	90.00	90.00	90.00	90.00
Ce_7O_{12}	Trigonal	90.00	90.00	90.00	90.00	120.00	120.00
CeO_2	Cubic	90.00	90.00	90.00	90.01	90.00	90.00
CeO_2	Cubic	90.00	90.00	90.00	89.99	90.00	90.00
Ce_2O_3	Cubic	90.00	90.00	90.00	90.00	90.00	90.00
Ce_2O_3	Trigonal	90.00	90.00	90.00	90.00	120.00	120.00
$Ce_{11}O_{20}$	Triclinic	96.17	96.14	90.12	90.09	99.71	100.00
Ce_3Ru	Orthorhombic	90.00	90.00	90.00	90.00	90.00	90.00
$CeRu_2$	Cubic	90.00	89.86	90.00	90.00	90.00	90.00
$Ce_{16}Ru_9$	Trigonal	90.00	90.01	90.00	90.07	120.00	120.00
Ce_4Ru_3	Monoclinic	90.00	92.67	114.96	111.82	90.00	88.40
Ce_7Ru_3	Hexagonal	90.00	90.00	90.00	90.00	120.00	120.00
$LaCeRu_4$	Cubic	90.00	90.03	90.00	90.01	90.00	90.03

Table S11 Various properties of N_2 on-top adsorption on $\sqrt{3} \times \sqrt{3} \times 6$ and $2 \times 2 \times 6$ Ru slabs calculated using the UNNP, as well as DFT and experimental values obtained in previous studies. Properties: $r_{\text{i-j}}$ = bond length between atoms *i* and *j*; *b* = buckling; $\varepsilon_{\text{vib},N_2}$ = vibrational energy; ν_{N_2} = wavenumber; ε_{ads} = adsorption energy; $\Delta\varepsilon_{\text{act,top-b-top}}$ = diffusion barrier for the on-top/bridge/on-top diffusion path; $\Delta\varepsilon_{\text{act},N_2\rightarrow 2N}$ = activation barrier dissociation energy for N_2 (on-top site) to 2N (closest hollow sites); and ε_{def} = deformation energy of the slab.

$\sqrt{3} \times \sqrt{3} \times 6\ (\theta = 1/3)$					
Property	DFT[27]	DFT(PW91)[28]	DFT(RPBE)[28]	UNNP	Exp.[27,28]
$r_{\text{N-N}}$ (Å)	1.11	-	-	1.13	1.10(4)
$r_{\text{Ru-N}}$ (Å)	2.00	-	-	1.96	2.00(5)
b (Å)	0.15	-	-	0.00	0.00(5)
$\varepsilon_{\text{vib},N_2}$ (meV)	-	-	-	273	274
ν_{N_2} (cm^{-1})	2239	-	-	2202	2195
ε_{ads} (eV)	-0.61	-	-	-0.43	-0.44
$2 \times 2 \times 6\ (\theta = 1/4)$					
Property	DFT[27]	DFT(PW91)[28]	DFT(RPBE)[28]	UNNP	Exp.[27,28]
$r_{\text{N-N}}$ (Å)		1.14		1.13	-
$r_{\text{Ru-N}}$ (Å)		2.02		1.95	-
ν_{N_2} (cm^{-1})		2229		2202	-
ε_{ads} (eV)	-0.47	-0.74	-0.40	-0.44	-0.31
$\Delta\varepsilon_{\text{act,top-b-top}}$ (eV)		0.45	0.51	0.56	-
$\Delta\varepsilon_{\text{act},N_2\rightarrow 2N}$ (eV)	1.92	-	-	1.63	-
ε_{def} (eV)		0.08	0.07	0.07	-

Table S12 N_2 adsorption end-on configurations on the top sites of the Ru_{153} hcp nanoparticle. DFT values from and UNNP values are provided for the adsorption energies ε_{ads}, vibrational frequencies ν_{N_2} and interatomic N-N distance $r_{\text{N-N}}$ (DFT | UNNP).

	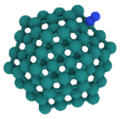	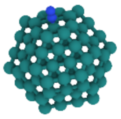	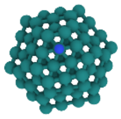	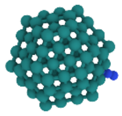
ε_{ads} (eV)	-0.357 | -0.289	-0.498 | -0.339	-0.554 | -0.258	-0.626 | -0.635
ν_{N_2} (cm^{-1})	2162 | 2163	2148 | 2147	2161 | 2171	2163 | 2175
$r_{\text{N-N}}$ (Å)	1.133 | 1.135	1.135 | 1.137	1.134 | 1.134	1.134 | 1.134
	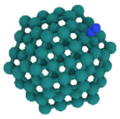	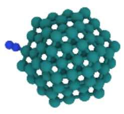	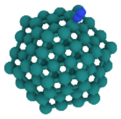	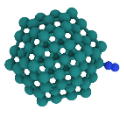
ε_{ads} (eV)	-0.724 | -0.631	-0.781 | -0.684	-0.796 | -0.777	-0.822 | -0.718
ν_{N_2} (cm^{-1})	2153 | 2175	2136 | 2165	2146 | 2153	2142 | 2152
$r_{\text{N-N}}$ (Å)	1.135 | 1.134	1.136 | 1.135	1.135 | 1.136	1.136 | 1.137
	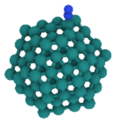	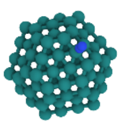	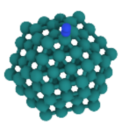	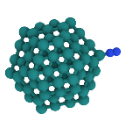
ε_{ads} (eV)	-0.844 | -0.797	-0.888 | -0.796	-0.890 | -0.8175	-1.031 | -0.904
ν_{N_2} (cm^{-1})	2145 | 2154	2140 | 2147	2139 | 2157	2143 | 2160
$r_{\text{N-N}}$ (Å)	1.136 | 1.136	1.136 |1.137	1.136 | 1.36	1.136 | 1.135

Table S13 Quantities used to calculate the average histograms for N_2 adsorption properties over various nanoparticle sizes based on the experimental particle size distribution from Ogura et al.[2] for $Ru/La_{0.5}Ce_{0.5}O_{1.75-x}$ catalysts reduced at 500 °C and 650 °C. N_{Ru} is the number of atoms in the ruthenium nanoparticle configuration, and d_F the average Feret diameter of the relaxed structure. The particle size categories correspond to those used to construct the experimental particle size distribution, and the weighting factors $w(T_{Red},d_F)$ correspond to the relative frequencies (see Fig. S9). $N_{NP}(d_F)$ is the number of particle configurations within each category.

N_{Ru}	d_F in nm	Category	$N_{NP}(d_F)$	$w(500\ °C,d_F)$	$w(650\ °C,d_F))$
63	0.995	0.5 nm - 1.0 nm	1	0.024	0.018
103	1.253	1.0 nm - 1.5 nm	1	0.24	0.40
153	1.511	1.5 nm - 2.0 nm	4	0.44	0.38
167					
238	1.771				
252					
323	2.030	2.0 nm - 2.5 nm	2	0.22	0.17
347					
625	2.539	2.5 nm - 3.0 nm	1	0.065	0.03
1021	3.070	3.0 nm - 3.5 nm	1	0.011	0.0
1557	3.592	3.5 nm - 4.0 nm	1	0.0	0.006

Table S14 Reduction degree x, number of defects $N_D = N_{La(D)+Ce(D)+O(D)}$, and energy differences $\Delta e_{D,i}^{SBNP}$ and $\Delta e_{D,i}^{SB}$ for a set of configurations with an SS cationic surface. The definition for the energy differences is provided in Data S4.

x	N_D	$\Delta e_{D,i}^{SBNP}$ (eV)	$\Delta e_{D,i}^{SB}$ (eV)
0.00	0	0.00	0.00
0.00	8	1.68	5.37
0.00	10	1.54	5.70
0.00	12	1.32	5.16
0.00	15	1.39	5.23
0.00	17	1.62	5.21
0.00	17	1.60	5.54
0.00	23	1.51	5.57
0.00	24	1.61	5.29
0.07	1	0.00	0.00
0.07	6	1.47	4.31
0.07	11	1.32	4.68
0.07	13	0.85	3.44
0.07	13	0.90	4.22
0.07	18	0.88	3.43
0.07	19	0.84	4.62
0.07	20	0.79	3.70
0.07	24	0.92	4.32
0.14	1	0.00	0.00
0.14	8	0.24	2.17
0.14	9	0.36	2.07
0.14	13	0.31	2.25
0.14	14	0.00	1.80
0.14	18	0.48	2.97
0.14	19	0.18	2.31
0.14	20	0.02	2.62
0.14	27	0.13	3.34
0.20	1	0.00	0.00
0.20	5	-0.06	1.75
0.20	7	0.28	2.61
0.20	10	-0.36	1.61
0.20	14	-0.21	1.55
0.20	14	-0.22	2.36
0.20	17	-0.18	1.62
0.20	20	-0.25	1.98
0.20	22	-0.14	2.23

Supplemental References

1. Keating, P.R.L., Scanlon, D.O., and Watson, G.W. (2014). The nature of oxygen states on the surfaces of CeO2 and La-doped CeO2. Chem. Phys. Lett. *608*, 239–243. https://doi.org/10.1016/J.CPLETT.2014.05.094.

2. Ogura, Y., Sato, K., Miyahara, S.I., Kawano, Y., Toriyama, T., Yamamoto, T., Matsumura, S., Hosokawa, S., and Nagaoka, K. (2018). Efficient ammonia synthesis over a $Ru/La_{0.5}Ce_{0.5}O_{1.75}$ catalyst pre-reduced at high temperature. Chem. Sci. *9*, 2230–2237. https://doi.org/10.1039/C7SC05343F.

3. Ogura, Y., Asai, T., Sato, K., Miyahara, S. ichiro, Toriyama, T., Yamamoto, T., Matsumura, S., and Nagaoka, K. (2020). Effect of Calcination and Reduction Temperatures on the Catalytic Activity of Ru/La0.5Ce0.5O1.75 for Ammonia Synthesis under Mild Conditions. Energy Technol. *8*, 2000264. https://doi.org/10.1002/ENTE.202000264.

4. Esch, F., Fabris, S., Zhou, L., Montini, T., Africh, C., Fornasiero, P., Comelli, G., and Rosei, R. (2005). Electron localization determines defect formation on ceria substrates. Science (80-.). *309*, 752–755. https://doi.org/10.1126/SCIENCE.1111568.

5. Kusada, K., Kobayashi, H., Yamamoto, T., Matsumura, S., Sumi, N., Sato, K., Nagaoka, K., Kubota, Y., and Kitagawa, H. (2013). Discovery of face-centered-cubic ruthenium nanoparticles: Facile size-controlled synthesis using the chemical reduction method. J. Am. Chem. Soc. *135*, 5493–5496. https://doi.org/10.1021/ja311261s.

6. Liu, L., Yu, M., Hou, B., Wang, Q., Zhu, B., Jia, L., and Li, D. (2019). Morphology evolution of fcc Ru nanoparticles under hydrogen atmosphere. Nanoscale *11*, 8037–8046. https://doi.org/10.1039/C9NR01611B.

7. Stiess, M. (2009). Mechanische Verfahrenstechnik : Partikeltechnologie. 1 (Springer Berlin Heidelberg).

8. Weber, S., Zimmermann, R.T., Bremer, J., Abel, K.L., Poppitz, D., Prinz, N., Ilsemann, J., Wendholt, S., Yang, Q., Pashminehazar, R., et al. (2022). Digitization in Catalysis Research: Towards a Holistic Description of a Ni/Al_2O_3 Reference Catalyst for CO_2 Methanation. ChemCatChem *14*, e202101878. https://doi.org/10.1002/CCTC.202101878.

9. Leitherer, A., Yeo, B.C., Liebscher, C.H., and Ghiringhelli, L.M. (2023). Automatic identification of crystal structures and interfaces via artificial-intelligence-based electron microscopy. npj Comput. Mater. 2023 91 *9*, 1–11. https://doi.org/10.1038/s41524-023-01133-1.

10. Wang, T., Hu, J., Ouyang, R., Wang, Y., Huang, Y., Hu, S., and Li, W.X. (2024). Nature of metal-support interaction for metal catalysts on oxide supports. Science (80-.). *386*, 915–920. https://doi.org/10.1126/SCIENCE.ADP6034.

11. Esch, B. Von Der, Peters, L.D.M., Sauerland, L., and Ochsenfeld, C. (2021). Quantitative comparison of experimental and computed IR-spectra extracted from ab initio nolecular dynamics. J. Chem. Theory Comput. *17*, 985–995. https://doi.org/10.1021/acs.jctc.0C01279.

12. Stukowski, A. (2010). Visualization and analysis of atomistic simulation data with OVITO-the Open Visualization Tool. Model. Simul. Mater. Sci. Eng. *18*, 015012. https://doi.org/10.1088/0965-0393/18/1/015012.

13. Heitjans, P., and Kärger, J. (2005). Diffusion in condensed matter: Methods, materials, models (Springer Berlin Heidelberg) https://doi.org/10.1007/3-540-30970-5.

14. Wang, Y., Kavanagh, S.R., Burgués-Ceballos, I., Walsh, A., Scanlon, D., and Konstantatos, G.

(2022). Cation disorder engineering yields AgBiS2 nanocrystals with enhanced optical absorption for efficient ultrathin solar cells. Nat. Photonics 2022 163 *16*, 235–241. https://doi.org/10.1038/s41566-021-00950-4.

15. Urban, A., Abdellahi, A., Dacek, S., Artrith, N., and Ceder, G. (2017). Electronic-Structure Origin of Cation Disorder in Transition-Metal Oxides. Phys. Rev. Lett. *119*, 176402. https://doi.org/10.1103/PHYSREVLETT.119.176402.

16. Abdellahi, A., Urban, A., Dacek, S., and Ceder, G. (2016). Understanding the Effect of Cation Disorder on the Voltage Profile of Lithium Transition-Metal Oxides. Chem. Mater. *28*, 5373–5383. https://doi.org/10.1021/ACS.CHEMMATER.6B01438.

17. Cowley, J.M. (1950). An approximate theory of order in alloys. Phys. Rev. *77*, 669–675. https://doi.org/10.1103/PhysRev.77.669.

18. Valadez Huerta, G., Nanba, Y., Kurata, I., Nakago, K., Takamoto, S., Shinagawa, C., and Koyama, M. (2021). Calculations of Real-System Nanoparticles Using Universal Neural Network Potential PFP. arXiv:2107.00963 [cond-mat.mtrl-sci]. https://doi.org/10.48550/arXiv.2107.00963.

19. Nanba, Y., Ishimoto, T., and Koyama, M. (2017). Structural Stability of Ruthenium Nanoparticles: A Density Functional Theory Study. J. Phys. Chem. C *121*, 27445–27452. https://doi.org/10.1021/acs.jpcc.7b08672.

20. Jain, A., Ong, S.P., Hautier, G., Chen, W., Richards, W.D., Dacek, S., Cholia, S., Gunter, D., Skinner, D., Ceder, G., et al. (2013). Commentary: the Materials Project: a materials genome approach to accelerating materials innovation. APL Mater. *1*, 011002. https://doi.org/10.1063/1.4812323.

21. Rivera Rocabado, D.S., Noguchi, T.G., Hayashi, S., Maeda, N., Yamauchi, M., and Ishimoto, T. (2021). Adsorption States of N2/H2Activated on Ru Nanoparticles Uncovered by Modulation-Excitation Infrared Spectroscopy and Density Functional Theory Calculations. ACS Nano *15*, 20079–20086. https://doi.org/10.1021/ACSNANO.1C07825.

22. Katoch, S., Chauhan, S.S., and Kumar, V. (2021). A review on genetic algorithm: past, present, and future. Multimed. Tools Appl. *80*, 8091–8126. https://doi.org/10.1007/S11042-020-10139-6.

23. Hinuma, Y., Toyao, T., Kamachi, T., Maeno, Z., Takakusagi, S., Furukawa, S., Takigawa, I., and Shimizu, K.I. (2018). Density Functional Theory Calculations of Oxygen Vacancy Formation and Subsequent Molecular Adsorption on Oxide Surfaces. J. Phys. Chem. C *122*, 29435–29444. https://doi.org/10.1021/ACS.JPCC.8B11279.

24. Zhang, X., Zhu, L., Hou, Q., Guan, J., Lu, Y., Keal, T.W., Buckeridge, J., Catlow, C.R.A., and Sokol, A.A. (2023). Toward a Consistent Prediction of Defect Chemistry in CeO2. Chem. Mater. *35*, 207–227. https://doi.org/10.1021/ACS.CHEMMATER.2C03019.

25. Chodera, J.D. (2016). A simple method for automated equilibration detection in molecular simulations. J. Chem. Theory Comput. *12*, 1799–1805. https://doi.org/10.1021/acs.jctc.5b00784.

26. Lee, E.M.Y., Ludwig, T., Yu, B., Singh, A.R., Gygi, F., Nørskov, J.K., and Pablo, J.J. de (2021). Neural network sampling of the free energy landscape for nitrogen dissociation on ruthenium. J. Phys. Chem. Lett. *12*, 2954–2962. https://doi.org/10.1021/ACS.JPCLETT.1C00195.

27. Mortensen, J.J., Morikawa, Y., Hammer, B., and Nørskov, J.K. (1997). Density functional calculations of N_2 adsorption and dissociation on a Ru(0001) surface. J. Catal. *169*, 85–92. https://doi.org/10.1006/JCAT.1997.1661.

28. Herron, J.A., Tonelli, S., and Mavrikakis, M. (2013). Atomic and molecular adsorption on Ru(0001). Surf. Sci. *614*, 64–74. https://doi.org/10.1016/J.SUSC.2013.04.002.
